# Alpha-synuclein overexpression reduces neural activity within a basal ganglia vocal nucleus in a zebra finch model

**DOI:** 10.1371/journal.pone.0333158

**Published:** 2026-07-16

**Authors:** Brian R. Dominguez, Gabriel Holguin, Madeleine S. Daly, Reed T. Bjork, Stephen L. Cowen, Julie E. Miller

**Affiliations:** 1 Department of Neuroscience, University of Arizona, Tucson, Arizona, United States of America; 2 Department of Psychology, University of Arizona, Tucson, Arizona, United States of America; 3 Evelyn F. McKnight Brain Institute, University of Arizona, Tucson, Arizona, United States of America; 4 Departments of Speech, Language and Hearing Sciences, Neurology, Graduate Interdisciplinary Program in Neuroscience, and the BIO5 Institute, Tucson, Arizona, United States of America; Louisiana State University Health Sciences Center, UNITED STATES OF AMERICA

## Abstract

Changes in vocal pitch, loudness, and timing are prevalent in Parkinson’s Disease (PD) and a target for early intervention and treatment. The neural mechanisms underlying these impairments are not understood, motivating work in animal models. The adult male zebra finch songbird is uniquely poised for these studies given vocally-dedicated brain nuclei and a quantifiable output (birdsong). Our prior publication revealed that injection of an adeno-associated virus (AAV5) expressing the human (h) alpha-synuclein (h*SNCA*, a-syn) gene into basal ganglia vocal nucleus Area X results in elevated insoluble a-syn protein and parkinsonian-like changes including softer, shorter, and reduced vocalizations compared to controls. Here, we test the hypothesis that AAV-h*SNCA* overexpression reduces the firing rate of specific neuronal sub-types in Area X using *in vivo* recordings in anesthetized finches. Five classes of neurons were differentiated in AAV-treated finches based on waveform width (narrow vs. wide) and firing rates (low vs. high). We found that neurons in the AAV-h*SNCA* group with wide waveforms exhibited reduced firing rates and enhanced post-peak rebound compared to AAV controls. No differences in firing rate nor waveform shape were detected for the narrow waveform neurons. Our findings provide the first characterization of early a-syn-driven neural activity changes in vocal control neurocircuitry.

## 1. Introduction

Pathological accumulation of alpha-synuclein (a-syn) is believed to contribute to multiple neurodegenerative diseases, including Parkinson’s disease (PD). Accumulation of toxic a-syn species culminates in the development of Lewy Bodies and Lewy Neurites that are detected in inherited and sporadic cases of PD, dementia with Lewy Body disease, and Multiple Systems Atrophy [1–5]. How abnormal increases in a-syn protein expression and its aggregation disrupts ongoing neuronal activity in the basal ganglia (BG) is not understood, yet this information is critical to developing cell-specific therapeutic targets [6–8]. One experimental approach is to examine how elevated a-syn protein interferes with the ongoing firing activity of striatal neurons.

Elevated levels of a-syn protein at cortico-striatal synapses can result in synaptic failure and loss of cortico-striatal plasticity based on electrophysiological recordings from rodent brain slices [6–11]. Striatal Medium Spiny Neurons (MSNs) show alterations in dendritic spine density, interactions between a-syn aggregates and their NMDA receptors, and reduced efficacy of cortical pre-synaptic glutamatergic input that affects their firing rate [12–15]. Furthermore, burst firing is a characteristic feature of MSNs [16], and dysregulated MSN burst firing may contribute to PD as shown in 6-hydroxydopamine (6-OHDA) models [17]. A recent study used induced human pluripotent stem cells from patients with Multiple System Atrophy of the Parkinsonian type that were differentiated into MSNs. These MSNs showed reduced spontaneous firing rates, frequency, and amplitude of miniature post-synaptic currents. These changes were also associated with increased a-syn release [18]. How *in vivo* cell-specific neuronal firing patterns in the basal ganglia relates to the behavioral output is unclear and a critical gap. The scant literature on *in vivo* recordings in rodent h*SNCA* overexpression models comes from nigral, thalamic, and hippocampal neurons evaluating movement and cognition [19–21]. Pairing electrophysiological changes in basal ganglia circuitry with vocal behavior offers an early and critical entry-point into furthering our understanding of the effects of alpha-synucleinopathies on neural circuits.

Voice and speech deficits are prevalent in PD and affect quality of life [22,23]. Early-stage vocal deficits (e.g., a soft, monotonous voice, altered speaking rate) can appear years before the movement symptoms, highlighting their utility as an early diagnostic tool, also with the support of machine learning algorithms and smartphone applications [24–37]. Our group and others have modeled PD-related vocal deficits using pharmacological and genetic approaches in rodents and songbirds with data from these experiments implicating the h*SNCA* gene in driving early vocal dysfunction [38–46].

**Fig 1:**
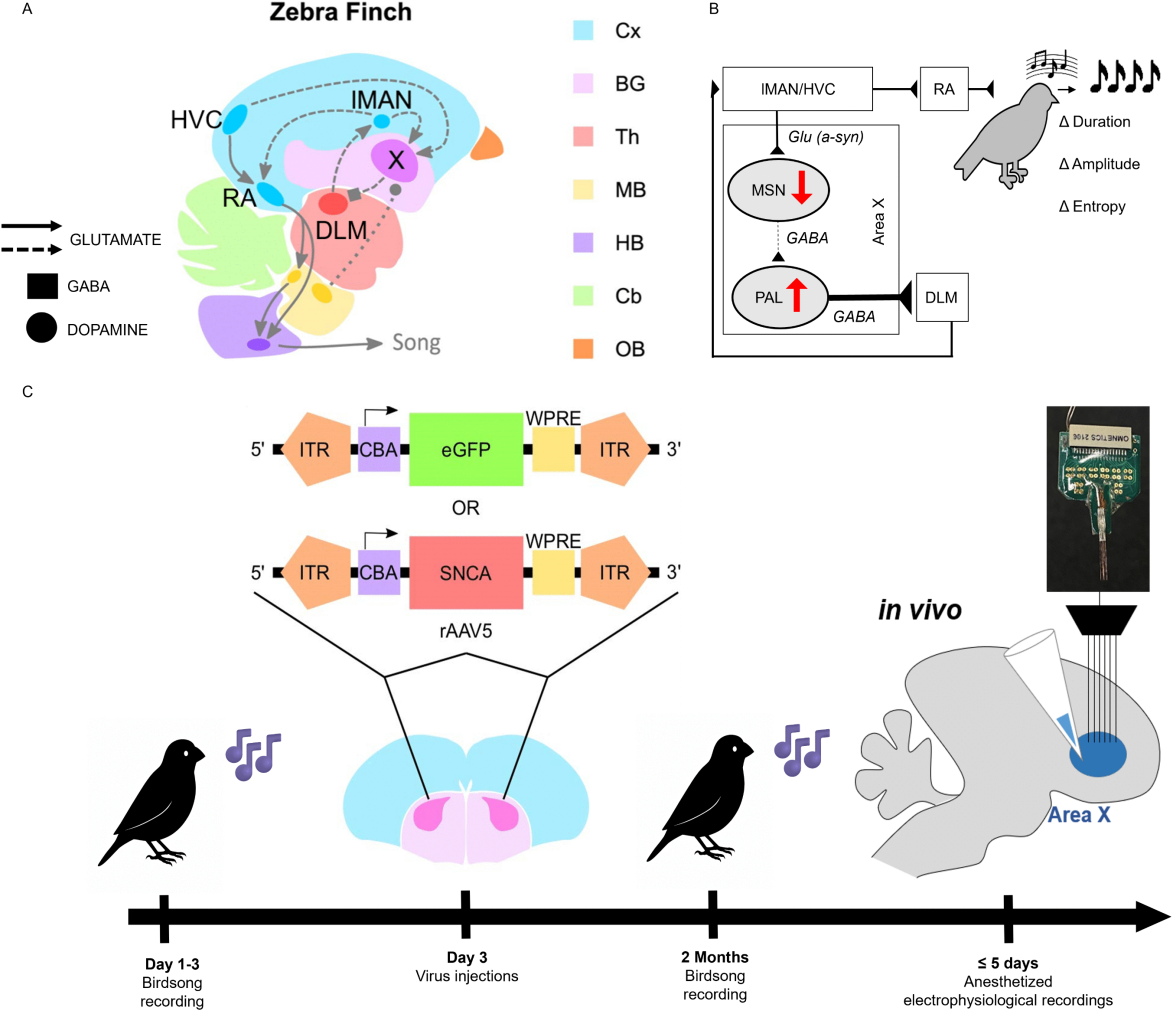
Song circuitry, hypothetical model, and experimental timeline. **A. Finch song control circuitry.** Brain nuclei in the adult male finch are specialized for song learning and production (HVC and Area X: proper names; RA: robust nucleus of the Arcopallium, lMAN: lateral magnocellular nucleus of the anterior nidopallium). The solid lines represent cortico-brainstem circuits involved in vocal production (finch: HVC to RA to brainstem). The dashed lines represent cortico-basal ganglia-thalamo-cortico circuits for on-going vocal learning and modification (lMAN-Area X-DLM-lMAN). Abbreviations: Cx – cortex. BG – basal ganglia. DLM – dorsal medial nucleus of the thalamus. Th – thalamus. MB – midbrain. HB – hindbrain. Cb – cerebellum. OB – olfactory bulb. Area X (violet) contains striatal, globus pallidus, and interneurons but in the human BG, striatal and pallidal areas are separate. Several auditory areas provide input to these vocal nuclei not shown here [60,61]. Figure and legend modified from finch brain image in Fig 1 of Medina et al. (2022). **B**. **Hypothetical model for a-syn driven changes in neural activity in Area X and consequences on song control circuitry.** Cortical vocal nuclei (lMAN, HVC) provide glutamatergic (Glu) input to Area X neurons. Striatal Area X MSNs provide GABAergic input to globus-pallidus like (GP, PAL) neurons including those that inhibit thalamic nucleus DLM. We *predict* that abnormally elevated levels of AAV-driven a-syn protein in Area X could alter intrinsic membrane properties of MSNs and/or reduce Glu input to MSNs. Consequently, MSNs would reduce their firing rate (red arrow), leading to increased GP PAL projection neuron firing and increased inhibition of DLM. Increased DLM inhibition could be responsible for changes in duration, amplitude (loudness) and entropy of the song syllables in finches with the AAV-h*SNCA* phenotype [45]. **C. Tetrode array and experimental timeline.** Schematic of adeno-associated viral (AAV) constructs and coronal section showing bilateral song nucleus Area X (purple) modified from Fig 3b in Medina et al. (2022). Song was collected within three days of the bilateral injection into Area X and then again at two months post-virus injection followed by tetrode recordings in anesthetized finches within five days, for a total experimental duration of approximately two and a half months. At the end of the recording morning (≤3 hrs), the finch was humanely euthanized, and the brain removed post-mortem for cryosectioning followed by immunohistochemistry to confirm AAV and a-syn protein expression along with tetrode placement.

Songbird models offer distinct advantages over rodents in exploring how the brain encodes sensory and motor information, shaping vocal learning and production in response to feedback [47]. Males of the zebra finch species sing but the females do not. The male finch song control system in the brain (**Fig 1A**) has similar neural organization, genetics, and physiology of the basal ganglia to mammalian species [48–50]. The song control system has strong similarities to human speech and auditory centers in the brain [51–57]. Vocal learning and on-going song modification in the zebra finch model can be investigated through the anterior forebrain pathway, a structure homologous to the cortico-basal ganglia-thalamo-cortical loop in humans and other mammals [50]. Song-dedicated brain region Area X in the basal ganglia shares similar genetics to human striatum, a major area involved in speech initiation and learning, and has cell types characteristic of mammalian neuronal populations [48,54,58,59].

Electrophysiological recordings in singing finches and anatomical tracings show that MSNs in Area X drive firing activity of globus pallidus-like (PAL) projection neurons [50,58,62]. As in mammals, GABAergic MSNs and Globus-pallidus (GP, PAL) type neurons receive glutamatergic (Glu) inputs from cortex (in finch-song nuclei HVC and lMAN, Fig 1A). GP neurons project to the dorsal medial thalamic nucleus (DLM) which then relays its output to the cortex [63]. While studies of MSN activity in animal models and humans are difficult due to low spontaneous activity [14,64], finch MSNs are dynamically active during singing and show temporally precise relationships to song structure [62,65]. DLM projections to HVC can also modulate syllable transitions [66]. High tonic firing in these PAL neurons inhibits DLM, reducing excitation onto lMAN and then subsequently onto song nucleus RA, leading to altered song output including variations in pitch [55,62]. Area X and lMAN neurons exhibit firing variability that is related to birdsong features [55,62]. The well-characterized neuronal populations in these brain nuclei and circuitry connections make this a powerful model to study the impact of alpha-synucleinopathies on vocalizations.

In our prior work, we showed that targeting Area X with an adeno-associated virus (AAV) driving increased expression of the human (h) wild-type *SNCA* gene leads to PD-like changes in the song including reduced duration and intensity [45]. We also showed that a-syn overexpression results in protein aggregates in Area X cell bodies [46]. Here, as a critical first step, we categorize cell-specific firing patterns and waveforms in Area X that are affected by a-syn overexpression compared to controls using tetrode recordings in anesthetized finches immediately following singing behavior. Our working hypothesis (Fig 1B) is that h*SNCA* overexpression in Area X will decrease firing rates and alter burst-firing in putative Area X MSNs. We tested this hypothesis by recording from Area X neurons in anesthetized zebra finches.

## 2. Materials and methods

### 2.1. Finch subjects, song recording and analyses

All animal use was approved by the Institutional Care and Use Committee at the University of Arizona through protocol approval number 13–489 to J.E. Miller. At the end of the experiments, finches were humanely euthanized with an overdose of isoflurane anesthesia. We complied with the ARRIVE guidelines (Animal Research: Reporting of In Vivo Experiments). Details on finch numbers, criteria for study inclusion/exclusion, and experimental endpoints are provided in the following sub-sections. Fig 1C shows our experimental timeline, workflow, and tetrode array.

Animal procedures followed Medina et al. (2022) and are summarized here. Only finches that showed normal eating/drinking, perching, and movement in their cages were selected for the study. Finches that did not sing prior to the pre-injection day were excluded from the study and returned to the general aviary population. Adult non-breeding male zebra finches (approx. 197–365 days post-hatch at time of euthanasia) were moved into individual sound attenuation chambers (Eckel Noise Control Technologies) and acclimated under a 14:10 h light-dark cycle for at least two days prior to song recordings and neurosurgery. Behavioral experiments were conducted in the morning from lights-on for two hours to match our prior study [45] along with ensuring singing-driven neuronal activity just prior to conducting the tetrode recordings in anesthetized finches.

**Song recordings and analyses:** Songs were acquired from individual males singing alone (known as undirected song) using free-field omnidirectional microphones (Shure 93) suspended at set points over the center of the cage, connected to an audiobox (Audiobox: 44.1 kHz sampling rate per 24-bit depth; Niles, IL) and recorded with Sound Analysis Pro 2011 (SAP 2011, [67] http://soundanalysispro.com/). Song data collected and used for this study can be found at the publicly accessible figshare site, University of Arizona ReData repository upon publication: doi.org/10.25422/azu.data.27868266.

A basic unit of birdsong is defined as a motif; a motif is comprised of a sequence of repeated syllables (S2A File **Fig Exemplar song motif and song syllable data).**

Wav files from the two-hour recording period were joined (Shuang’s audio joiner) and then the entire file was viewed in Audacity (https://www.audacityteam.org/). The most frequently occurring motif was then identified in the joined wav file for two pre-surgical mornings (time point 0) and then two post-surgical mornings (time point, 2 months, Fig 1C) by an investigator blind to the AAV condition. The two-month timepoint was selected as the experimental endpoint for song analyses and tetrode recordings in a non-survival surgery followed by humane euthanasia. Our prior publications show that the most prominent song and protein changes occurred at this time point in the AAV-h*SNCA* finches [45,46]. Following morning lights-on, the first motif was identified in the wav file and then 25 consecutive renditions of the individual syllable wav files were exported from Audacity from the four mornings. Up to five unique syllables within a motif for each bird were included in the analyses.

Syllable wav files were processed in Matlab R2014 using the SAP SAT Tools box using code (provided by Dr. Nancy Day, Whitman College) to extract individual acoustic features as a feature batch function. Raw amplitude (intensity) measurements were calibrated based on sound chamber location as in our prior study [68]. Mean, standard deviation, and coefficient of variation (CV, standard deviation/mean) were then calculated by averaging 50 scores (25 syllable copies x 2 recording sessions) across time points 0 (pre-AAV injection) and 2 months post-AAV injection for each syllable within the bird’s motifs to account for natural, day to day fluctuations in song. For group level comparisons, a normalization score was obtained per bird by dividing timepoint two months (2) post-injection by pre-injection (0). After verifying that scores were not normally distributed, scores were then compared using the Mann Whitney U test in GraphPad Prism (version 10) between AAV-h*SNCA* and AAV-GFP groups for all syllable types combined and then sorted by syllable type (S1 File. **Table 1. song data and statistics**) given prior findings on a-syn differential effects [45]. Syllables were sorted into noisy, mixed, or harmonic categories based on visual inspection and Wiener Entropy (WE) scores following our prior publications (S2A File. **Fig Exemplar song motif and song syllable data).**

[45,69]. We focused our analyses on comparing duration, amplitude, and WE scores between the two AAV groups, and their correlations with the electrophysiological analyses for an individual bird (see section on **Statistics** and S2–S3 File. **Figs Exemplar song motif and song syllable data and No significant correlation between song features and firing rate).**

A subset of these birds was used in our separate publication evaluating regional distribution of a-syn protein in finch song nuclei and linear correlations to syllable features in Area X and lMAN [46].

As described in Medina et al. (2022), duration is the length of each syllable (in seconds), and amplitude is the intensity or loudness of the syllable (in decibels-dB). WE is a measure of uniformity in the power spectra (i.e., structure of the syllable), where scores are calculated on a logarithmic scale; noisy syllables have values approaching zero while harmonic syllables have more negative scores. The coefficient of variation (CV) is calculated by dividing the standard deviation of 50 syllable renditions by the mean score and represents variability across motifs for an acoustic feature, modulated by the lMAN-Area X-DLM-lMAN loop [70].

### 2.2. Surgical procedures: viral injections

A total of 19 finches were originally used for adeno-associated virus (AAV) bilateral injections into Area X (n = 11, AAV5-CBA-h*SNCA* [human alpha-synuclein]; n = 8, AAV5-CBA-eGFP controls [Green Fluorescent Protein]; CBA: chicken beta-actin promoter). One bird in each group spontaneously died prior to the two month post-AAV injection experimental endpoint due to unknown causes and therefore, they were removed from the study. We alternated the order of the AAV experimental vs. control virus surgeries when two surgeries were done on the same day.

The surgery suite and instruments were prepared for aseptic survival surgery under PI Miller’s approved IACUC Protocol 13–489. Following two hours of morning song collection, finches were put under isoflurane anesthesia with oxygenation (2–3%, 0.5 liters/min oxygen) and set up in the stereotaxis. The analgesic lidocaine (0.05 ml) was subcutaneously injected across the planned incision site of the scalp at four locations. For the bilateral injections into Area X, stereotaxic coordinates were used from the bifurcation of the mid-sagittal sinus: 3.1 mm rostral-caudal, 1.62 mm medio-lateral, a depth of 3.1 mm, and head angle of 40 degrees. These coordinates avoid indirect targeting of song nucleus lMAN with virus as previously described [45]. Viruses (AAV5) were obtained from the University of North Carolina Viral Vector Core through financial support of the Michael J. Fox Foundation for Parkinson’s Research (https://www.michaeljfox.org/research-tools).

A glass pipette was fitted into a Nanoject II pressure-injector and back-filled with mineral oil, then loaded with either AAV. Approximately 500 nL of virus was injected at a rate of 27.6 nL/injection every 15 seconds for a total of 18 injections followed by five minutes of wait time then inspection of pipette tip for clogging. Upon removal of isoflurane anesthesia, birds were monitored for any signs of pain and distress while on a homeothermic heating blanket. Within a half-hour, birds were noted to be alert, upright, and vocalizing and did not require any post-operative analgesia. They were returned to their sound chambers and monitored for any surgical complications, abnormal postural or movement, eating and drinking behavior for at least three days post-surgery by research personnel fully trained in bird care. Their drinking water was supplemented with an oral antibiotic dose of TMS (Sulfamethoxazole and Trimethoprim Oral Suspension, USP grade; 1 ml/100 ml deionized water). University Animal Care staff performed daily checks on animal welfare with Miller lab personnel providing oversight throughout the duration of the experiment.

### 2.3. Tetrode Implantation and Electrophysiology

**Fig 2:**
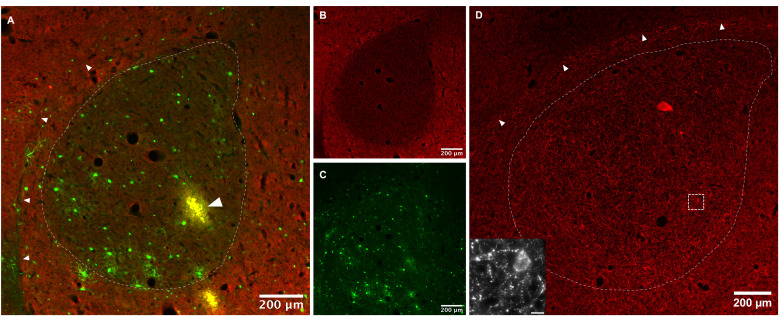
AAV expression in finch song nucleus Area X. The Area X region is represented by the dotted line. Small arrowheads denote the border between the striatum and the nidopallium. Staining for alpha-synuclein (a-syn) protein is shown in red. **A)** An AAV control expressing finch has GFP labeled cell bodies (green) in Area X, and the large arrowhead denotes the last recorded tetrode position (yellow). **B)** A-syn protein expression is low in Area X compared to surrounding basal ganglia in this AAV-GFP finch. **C)** View of (A) showing the GFP channel only. **D)** An AAV-h*SNCA* expressing finch shows widespread expression of a-syn in processes throughout Area X. Inset in bottom left corner shows a zoomed in view of bead-like a-syn protein in the processes (inset scale bar = 10 µm).

**Surgical procedure and implantation:** Approximately two months post-virus injection, 17 finches, out of the 19 originally included (two birds died post-virus injection and prior to tetrode implantation), were anesthetized with isoflurane (2–3%, constant flow rate of 0.5–0.8 L of oxygen/min). While under anesthesia, they underwent acute electrophysiological recordings of right hemisphere Area X as a non-survival surgery approved under PI Miller’s IACUC Protocol #13–489. Breathing rate was monitored throughout the experiment, and an infrared heating pad (Kent Scientific) maintained body temperature at 37ºC. The same stereotaxic coordinates were used for the AAV injections targeting Area X. **Ground wire insertion:** A ground/reference wire was inserted into the left hemisphere, contralateral to the virus injection site (mid-sagittal sinus: 3.1 mm rostral-caudal, 1.62 mm medio-lateral). After performing the craniotomy, the dura was pierced with a 30G needle, and the end of the ground wire was lowered to a depth of 2–3 mm. Gel superglue was used to secure the wire to the acid-etched skull (acid from the Metabond kit). Zip Kicker (Pacer Technology) was used to rapidly cure the superglue. **Electrode array insertion:** The existing craniotomy on the right hemisphere (used for virus injection) was expanded to a diameter of ~1.5 mm to better accommodate the electrode array and to have access to dura that was undamaged during virus injection. Tetrode position was confirmed in Area X post-mortem following electrophysiological recordings (Fig 2).

**Electrophysiological recordings:** Neurons from Area X were recorded using a custom-made 800 µm diameter tetrode array composed of 8 twisted-wire tetrodes. Tetrodes were constructed from polyamide-insulated nickel chromium steel wire (~12 µm in diameter; see Fig 1C). Prior to insertion, tetrodes were coated with DiI to allow anatomical tracing of the electrode path (Fig 2A). Using a Micro-Positioning Controller (National Aperture, Inc.; MC-5B), the tetrode array was lowered in ~100 µm increments until the tip reached Area X at ~3100 µm (right hemisphere) and allowed to stabilize for 5–10 minutes prior to recording. Neural data was acquired at 20 kHz using the INTAN neural recording system (Intan Technologies, Inc.). Neural recordings from Area X neurons occurred in 5-minute blocks. After each 5-min block of data was acquired, the electrode array was lowered by depth ~50 µm and another recording block was acquired. The electrode was not moved in the medio-lateral direction. All recordings were restricted to Area X (3100–3300µm). Electrophysiological recordings used for this study can be found at the publicly accessible figshare site, the University of Arizona ReData repository upon publication: doi.org/10.25422/azu.data.27868266

### 2.4. Spike-sorting procedures

Data was spike sorted using freely available software, Kilosort v2.5 (https://zenodo.org/records/4482749) [71] and Phy (https://github.com/cortex-lab/phy). Cluster quality was assessed by 1) viewing the inter-spike-interval plots and autocorrelograms (see S4 File. **Fig Individual waveforms and autocorrelograms**) to identify clusters with contamination (indicated by ISIs that were too small to be physiological). We also applied a criterion whereby neurons where >5% of ISIs were <1.5ms were eliminated. 2) During spike sorting, cross-correlograms were inspected to determine if there was a strong peak at lag = 0 which would suggest that the two clusters need to be merged.

**Cell-type estimation:** A detailed description of how cell-types were estimated is presented in the Results section. Briefly, neurons were segmented into five classes based on waveform width (half width and trough-to-peak width) and firing rate (S5 File. **Fig Waveforms, firing rates, and classification**).

### 2.5. Data/statistical analysis

All single-unit data was analyzed using Matlab (R2025a). All Matlab analysis code is provided in the Supplemental Material as S10 File. **Matlab code for ephys.** This folder contains the MATLAB files used by Co-Author Cowen to analyze the electrophysiological recordings.

Out of the 17 finches implanted with the tetrode array, four finches in the ASYN group yielded no recordings (e.g., neurons were quiet), and one GFP control finch showed no virus expression detected in Area X post-mortem. These five finches were therefore excluded from our data analyses but humanely euthanized with the other finches at the end of that day’s electrophysiological recording period. Thus, n = 12 finches (n = 6/per AAV group) were used for electrophysiological and song analyses presented in the Results section.

**Measurement of burst activity:** The temporal structure of the firing activity of individual neurons was quantified using “local variance” (LV), a measure that assesses the variability of inter-spike intervals (ISIs) and is robust to changes in firing rate [72]. LV is computed from the vector of ISIs for each neuron as shown in the formula below:


$$\begin{array}{c} LV=\frac{\text{3}}{n-\text{1}}\sum_{i=\text{1}}^{n-\text{1}}\left(\text{1}+\frac{\text{4}I_{i}I_{i+\text{1}}}{{{(I}_{i}+I_{i+\text{1}})}^{\text{2}}}\right)\left(\text{1}-\frac{\text{4}R}{I_{i}+I_{i+\text{1}}}\right), \end{array}$$ (1)

where $I_{i}$ and $I_{i+\text{1}}$ are the i-th and i+1 ISI and n is the number of ISIs. LV scores ~0 indicate tonic firing, scores ~1 show Poisson firing, and scores >1 indicate burst-like firing patterns.

Parametric and non-parametric tests were used for hypothesis testing. For group-level analysis of firing-rate data (n = 201 categorized neurons from n = 12 finches) non-parametric tests were used as firing rates do not follow normal distributions [73]. Consequently, rank-sum tests were used for two-group comparisons and the Kruskal-Wallis test was used for multi-group data. The Holm-Bonferroni correction was used to correct for multiple comparisons. For normally distributed data, ANOVA and t-tests were used followed by Tukey or Holm-Bonferroni post-hoc corrections. Alpha was set to 0.05. Relationships between neural data and each bird’s song features (e.g., mean Duration, Amplitude, and Entropy) were investigated using a generalized linear regression model where the outcome variable was neural activity (e.g., firing rate) and the independent variables were song features and group category (ASYN or GFP) as described in **Results and** S3 File. **Fig No correlation between song and firing rate**. Multicollinearity between independent variables can impair the ability to interpret regression models. We observed that some song features did indeed covary (r > 0.4 for 5 song feature pairs). To address this, we repeated the regression using the first three principal components of the independent variable matrix and compared results to those from the original regression model. As reported in S3 File**. Fig No correlation between song and firing rate**, similar results were obtained from the original and dimensionality-reduced model.

### 2.6. Tissue processing and immunohistochemistry

Immediately following the end of the neural recording session, finches were humanely euthanized by isoflurane inhalant overdose and transcardially perfused with warmed saline followed by chilled 4% paraformaldehyde in Dulbecco’s Phosphate Buffer Saline. Fixed brains were cryoprotected in 20% sucrose overnight then cryosectioned in the coronal plane at 30 µm on a Microtome cryostat through Area X. Tissue was processed, following our previously published methods [45,46] and summarized here: Hydrophobic borders were drawn on the slides, using a pap pen (ImmEdge, Vector Labs) followed by 3 X 5 minute washes in TBS with 0.3% Triton X (Tx). To block non-specific antibody binding, the tissue was then incubated for one hour at room temperature with 5% goat serum (Sigma #G-9023) in TBS/0.3% Tx then 3 x 5-minute washes in 1% goat serum in TBS/0.3% Tx were performed. A primary antibody was used against a-syn (1:250, rabbit, Proteintech 10842–1-AP, RRID: AB_2192672). GFP expression was robust enough to be visualized without an antibody.

The primary antibody was incubated in a solution of 1% goat serum in TBS/0.3% Tx overnight at 4 °C. One tissue section per slide was used as a ‘no primary’ control and a pre-absorption control experiment was performed to validate antibody specificity in a companion study evaluating the distribution of a-syn protein following AAV injection [46]. The next day, sections were washed 5 x 5 minutes in TBS/0.3% Tx and incubated for three hrs at room temperature in a fluorescently conjugated secondary antibody (1:1000: goat anti-rabbit 647 A-21245, RRID:AB_2535813). After secondary incubation, sections were washed 3 x 10 minutes in TBS followed by 2 x 5 washes in filtered TBS. Slides were then cover-slipped in Pro-Long Anti-Fade Gold mounting medium (Molecular Probes, P36930). Tissue was imaged using a Zeiss Axio Observer 7 with Apotome III Microscope under the University of Arizona Imaging Cores – Optical Core Facility (RRID:SCR_023355). Microscopy images used for Fig 2 can be found at the publicly accessible figshare site, the University of Arizona ReData repository upon publication: doi.org/10.25422/azu.data.27868266.

## 3. Results

### 3.1. Virally-driven expression is confirmed in song nucleus Area X

Fig 2 is a representative example of AAV expression in Area X. Two months post-virus injection, we detected GFP cell bodies in Area X (Fig 2A, C) accompanied by low a-syn protein expression (Fig 2B). By contrast, a finch overexpressing a-syn showed staining in processes and cell bodies (Fig 2D). These results are consistent with our two prior publications [45,46].

### 3.2. Extracellular identification of neuronal subtypes in anesthetized finches

Area X neurons share much in common with neurons in the mammalian striatum and pallidum. For example, anatomical evidence suggests that the most abundant Area X neuron type is homologous to the mammalian medium spiny neuron (MSN, [58]), with Area X and mammalian MSNs sharing wide waveforms, low firing rates, and bursting activity [74–76]. There is also evidence that Area X contains striatal fast-firing interneurons, cholinergic interneurons, and low-threshold spiking neurons [48,74,77]. Furthermore, Area X contains two classes of fast-firing pallidal-like neurons: local-circuit (HF-1) and thalamus-projecting (HF-2) [59,78]. In these studies, cell classes were identified by their firing rates and waveform features using multiple approaches from this literature, including correlating neuronal activity *in vivo* with birdsong and patch-clamp recording in brain slices. Our present study is constrained by the fact that all data was acquired from extracellular electrodes implanted in finches anesthetized using isoflurane. In mammals, isoflurane generally reduces firing rates (reviewed in [79,80], but the effects vary. A further constraint of our study was that recordings for a given neuron lasted five minutes. This is important as Goldberg and Fee identified putative MSN neurons in awake and non-singing finches that fired at rates as low as 0.01 Hz(74). Such low rates would make their detection in our experiment impossible. Consequently, it must be emphasized that mapping the five cell types identified here to cell types identified in awake finches must involve some caution.

We found no prior publications with recordings from Area X of adult anesthetized finches; prior work includes some anesthetized recordings from lMAN and Area X in the context of juvenile song learning [81]. Consequently, our classification was informed by criteria developed from *in vivo* recordings from Area X in awake finches and restricted to waveform shape, firing rate, and assessment of bursting activity in the following five identified categories [74,78,82]. Consequently, we computed the firing rate and spike width (half width and trough-to-peak width) for each neuron. The scatterplots and histograms of these variables can be seen in S5 File. **Fig Waveforms, firing rates, and classification**. The individual wave forms and autocorrelograms for all neurons in the study are found in S4 File **Fig Individual waveforms and autocorrelograms**. Unlike reports from awake finches, the firing rate distribution in the anesthetized finches was not bimodal, being instead long tailed and continuous with many neurons firing at low rates. This effect, which could be due to anesthesia, complicated the determination of a cutoff threshold for firing rate for cell-type identification. That said, we attempted to establish reasonable criteria for estimating cell types as described below.

**Fig 3:**
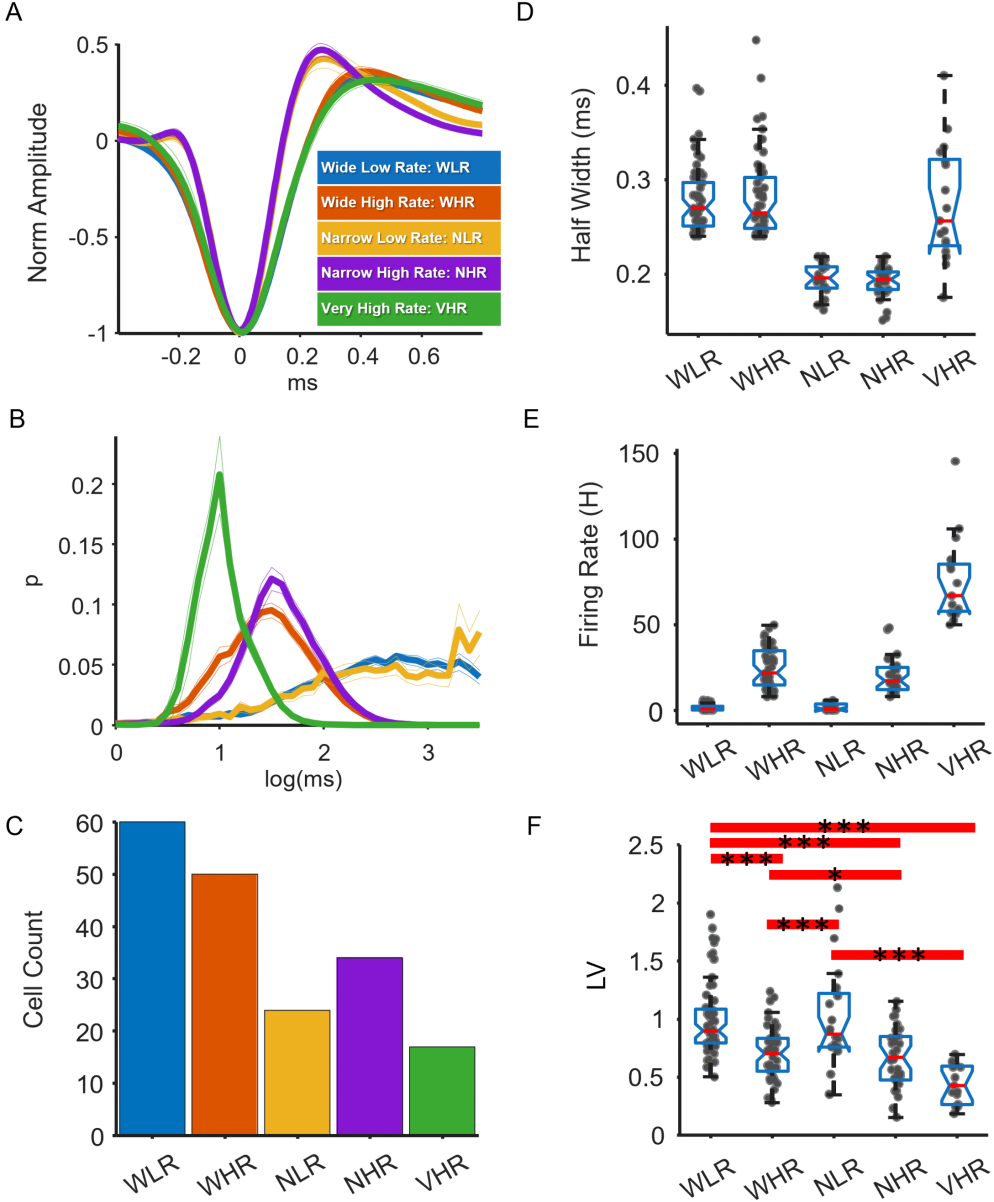
Identification of five classes of Area X neurons in anesthetized finches. **A)** Mean shape for each of the five identified classes of neurons (n = 185 neurons from n = 12 finches). Neurons were categorized based on spike width and firing rate (see Results). Amplitude is normalized such that the minimum (trough) = −1. Colors indicate neuron subtype (see key). **See** S4 File. **Fig Individual waveforms and autocorrelograms** for all waveform traces. **B)** Inter-spike interval histograms for each neuronal subtype. Y-axis indicates probability (p). **C)** Histogram indicating the number of identified neurons in each category. **D)** Box plot of the half-width (ms-milliseconds) for each neuronal group. Each dot indicates an individual neuron. Horizontal red lines indicate the median. Hypothesis tests were not performed as half-width was a key criterion for classification. **E)** Firing rates for each neuronal group (H-Hertz). Each dot indicates an individual neuron. Hypothesis tests were not performed as firing rate was a key criterion for classification. **F)** Burst-like firing differed between groups. While neurons were not categorized based on burst-firing, the measure of bursting (Local Variance-LV) indicated that Wide-Low-Rate neurons had increased burst-like firing (LV > 1) relative to other groups (ANOVA p < 0.000001, Tukey post-hoc comparison). Thick red lines indicate significant post-hoc differences (*p < 0.05, **p < 0.01 (not shown), ***p < 0.001). The red lines in the box plots indicate the median and the whiskers indicate 1.5X the Interquartile Range.

Neuron categorization and criteria (results shown in Fig 3, S4 File. **Fig Individual waveforms and autocorrelograms,** **S5 File****. Fig Waveforms, firing rates, and classification,** **S6 File**. **Fig Cluster separation between neuronal categories**):

1) **Wide-Low-Rate (WLR) Criteria**: Neurons firing ≤ 6 Hz with a peak half width > 0.25 ms or a peak-to-trough width of > 0.35 ms (Fig 3 **A,D,E**). Choosing a rigid threshold was difficult from our data as there was no clear bimodality in the spike-width distributions (S5 File. **Fig Waveforms, firing rates, and classification**) and while previous studies have determined firing rate thresholds, these studies were performed in awake and not anesthetized finches. Given the evidence that anesthesia, on average, lowers firing rates, we chose a threshold (6 Hz) that was below the 10-Hz threshold reported in singing birds [74]. However, as described above, putative MSNs in awake, non-singing birds may fire well below 1 Hz. While our finches are singing for a two hour period immediately prior to anesthesia and tetrode recording, we do not know the impact of anesthesia. Consequently, we will refrain from making strong claims that these are MSNs.
  Following classification, the firing properties of the WLR neurons were analyzed, revealing that WLR neurons were more ‘bursty” relative to other neuronal types (Fig 3F) as measured by Local Variance (LV, see Methods [83]). WLR neurons were also recorded the most out of the five identified cell types (Fig 3C). Still, considering the important caveats involved in inferring cell type from extracellular measures in anesthetized animals, the WLR cells shared key features of MSNs such as exhibiting wide waveforms, expressing relatively low firing rates, and expressing bursting activity [74–76].
2) **Wide-High-Rate (WHR) Criteria**: Neurons with a firing rate between 8 and 50 Hz with a peak half width > 0.25 ms or a peak-to-trough width of > 0.35 ms (Fig 3A,D,E). Post-classification analysis also showed that these neurons fired tonically (LV < 1, see Fig 3F). Tonically active WHR cells have features consistent with striatal cholinergic interneurons [74].
3) **Narrow-Low-Rate (NLR) Criteria**: Narrow waveform neurons firing ≤ 6 Hz [74] having a peak half width < 0.25 ms or a peak-to-trough width of < 0.35 ms (Fig 3A,D,E).
4) **Narrow-High-Rate (NHR) Criteria**: Narrow waveform neurons with rates ≥ 8 Hz but < 50 Hz (Fig 3A,D,E). Narrow waveforms are characteristic of local circuit fast-firing inhibitory neurons.
5) **Very-High-Rate (VHR) Criteria**: Neurons firing ≥ 50 Hz (Fig 3A,D,E). Area X pallidal-like projection neurons are characterized by exceedingly high firing rates [74,78]. Consequently, we established a category for neurons with rates ≥ 50 Hz. Waveform features were not used to further subdivide neurons in this group given its small size (n = 18 neurons, Fig 3C). Analysis of VHR firing activity revealed that these neurons exhibited decidedly tonic activity, with a mean LV of 0.51 (Fig 3F). These data suggest that the identified VHR neurons are type-2 pallidal-like projection neurons (HF-2 in [78,84]) given the presence of tonic firing and relatively wide waveforms. Furthermore, the small size of this group is consistent with an anatomical study that suggests PAL neurons comprise approximately just 5.4% of the Area X cell population [58] although in awake, singing birds, they appear to comprise a higher percentage of actively firing cells as identified using extracellular recordings [74,78].

It should also be noted that using the above criteria resulted in a set of ‘uncategorized’ neurons (S4 File. **Fig Waveforms, firing rates, and classification**) with >5% of ISIs < 1.5ms. Using this more stringent criterion, we eliminated 4.6% of neurons (n = 16). We chose not to analyze data from these cells in the main figures and in the other Supplemental figures given the difficulty in making reasonable inferences about their cell type.

### 3.3. Reduced Area X firing rates in ASYN overexpressing finches

**Fig 4:**
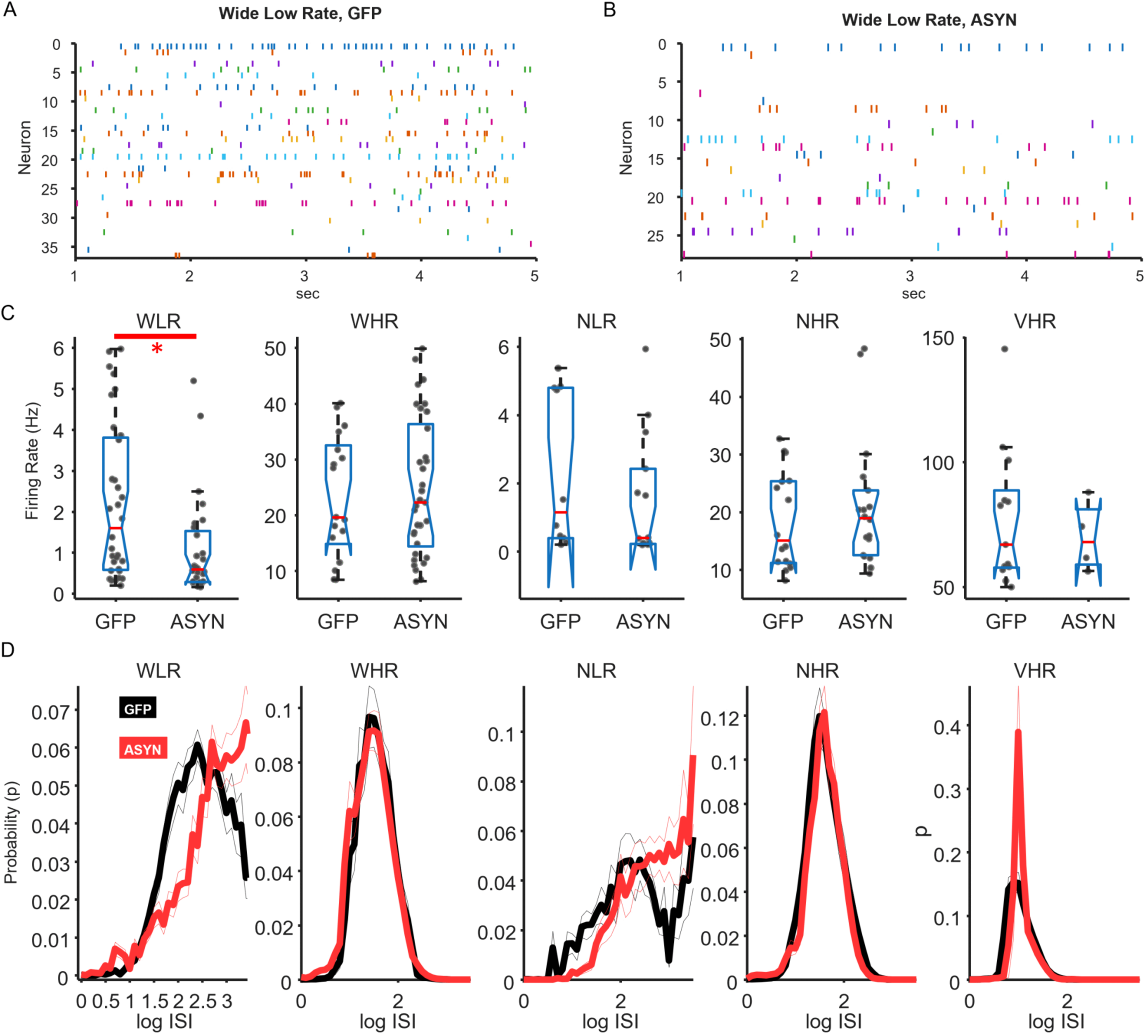
Reduced firing rates of WLR neurons in the ASYN group. **A-B)** Raster plots of Wide-Low-Rate (WLR) in the GFP and AAV-h*SNCA* (ASYN) groups. The x-axis shows time in seconds. Each row is a raster for a distinct neuron. **C)** Between-group comparison (GFP vs. ASYN) for each of the five neuron types. Wide-Low-Rate (WLR), Wide-High-Rate (WHR), Narrow-Low-Rate (NLR), Narrow-High-Rate (NHR), Very-High-Rate (VHR). WLR neurons were only found in 4/6 finches. Firing rates for WLR neurons were lower in the ASYN group relative to the GFP control (rank-sum test, p = 0.006 prior to correction, *p = 0.029 after Holm-Bonferroni correction). **D)** The mean inter-spike-interval (ISI) distribution for the ASYN and GFP groups (mean ±SEM).

We predicted in our model (Fig 1B) that a-syn expression would reduce WLR neuron (putative MSN) activity, thus releasing VHR neurons (putative PAL) from inhibition. Consequently, we investigated whether firing rates of WLR neurons were reduced in the ASYN group and whether rates in VHR neurons were increased. Visual inspection of raster plots of WLR activity (Fig 4A-B) suggested reduced WLR firing activity in the ASYN group. Statistical comparison of firing rates of GFP and ASYN neurons identified a significant difference (Fig 4C, rank-sum, p = 0.006 before correction and p = 0.029 after Holm-Bonferroni correction). No significant between-group differences were identified in the other neuronal classes, including the VHR neurons. S7 File. Fig **Individual animal variance** shows the within animal variance.

### 3.4. Burst firing was not changed in ASYN overexpressing finches

**Fig 5:**
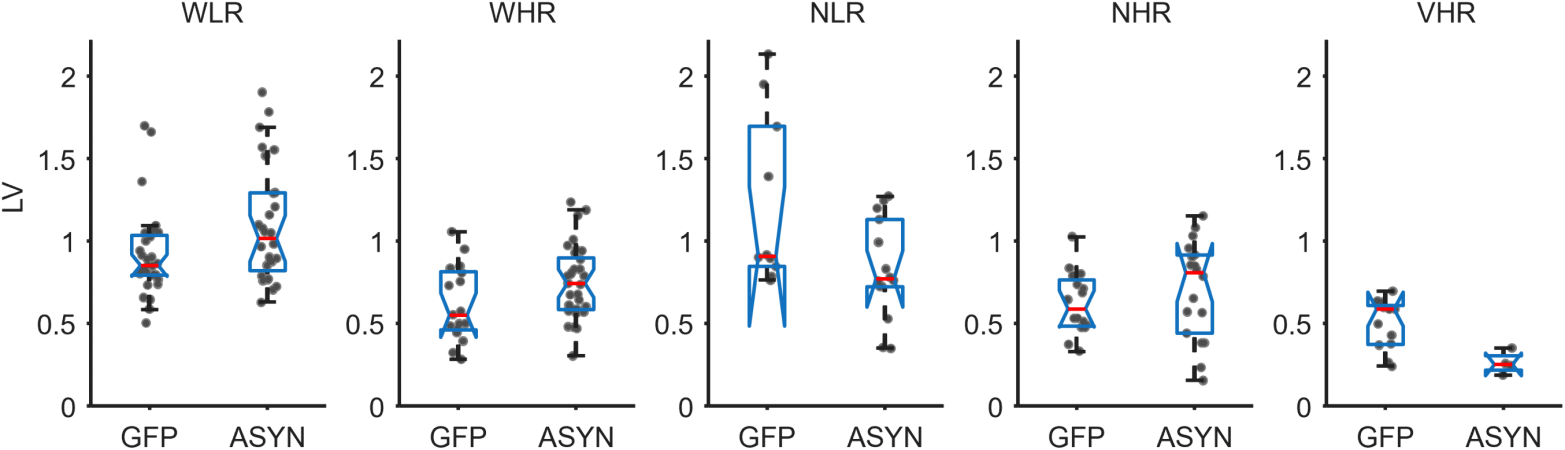
Local Variance (LV), a measure of bursting, is not different between ASYN and GFP control groups. Between-group comparisons (GFP vs. ASYN) were made for each of the five neuron types, and no between-group differences were identified in any of the neuron classes (rank-sum test, all p > 0.05 after Holm-Bonferroni post-hoc correction). Wide-Low-Rate (WLR), Wide-High-Rate (WHR), Narrow-Low-Rate (NLR), Narrow-High-Rate (NHR), Very-High-Rate (VHR).

While burst firing is a canonical property of MSNs, MSN burst firing may become dysregulated in PD. Indeed, altered MSN burst firing has been reported in the 6-OHDA mouse model of PD [85], non-human MPTP primate models [86], and in human PD patients [64]. Consequently, we predicted that burst firing will be affected by ASYN overexpression in finch Area X. To investigate this, the local variance (LV) measure [72], see Methods) was used to evaluate the pattern of inter-spike intervals and quantify the degree that neuronal activity was either tonic (low LV), Poisson (LV near 1), or bursty (LV > 1). Contrary to our prediction, analysis of spiking statistics with LV did not identify any difference between GFP and ASYN groups in LV values for any neuronal subtype (Fig 5, t-test, all p > 0.05 after Holm-Bonferroni post-hoc correction). Thus, while ASYN significantly reduced firing rates of WLR neurons, these changes were not accompanied by a change in burst-firing. See also S8 File. Fig **Rasters for cell types sorted by LV**.

### 3.5. Extracellular waveform shape was altered in wide-waveform neurons

**Fig 6:**
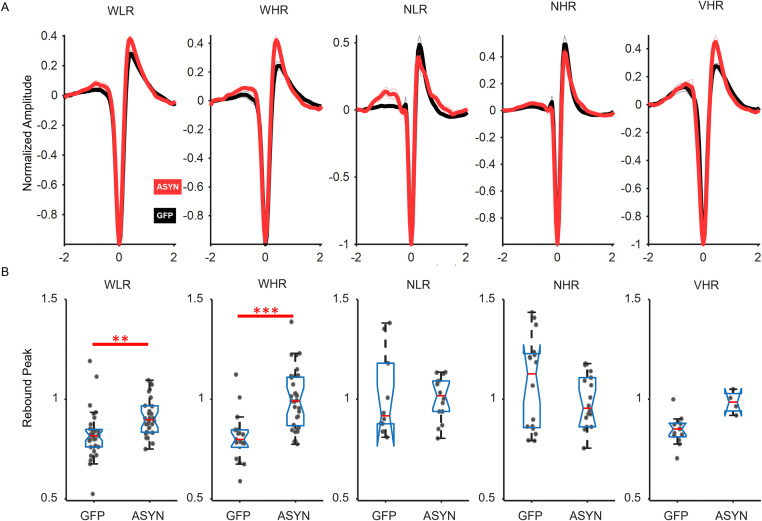
ASYN group shows enhanced post-peak rebound for wide-waveform neurons. **A)** Mean waveforms for the ASYN and GFP groups for each neuron type. For each waveform, data was normalized such that the distance from baseline to the trough = −1. Inspection of these plots suggested that wide-waveform (WLR, WHR) ASYN neurons had a larger rebound following the initial trough. **B)** Statistical analysis of the rebound was performed by measuring the normalized amplitude of the largest peak following t = 0 (rebound peak) for each neuron (dots). These values were compared between groups using rank-sum tests and p values were adjusted using the Holm-Bonferroni correction. **p < 0.01, ***p < 0.001. Error bars in box plots indicate ±SEM. Wide-Low-Rate (WLR), Wide-High-Rate (WHR), Narrow-Low-Rate (NLR), Narrow-High-Rate (NHR), Very-High-Rate (VHR). See also S9 File. **Fig Supplemental to** Fig 6 **individual waveforms.**

While waveform features and firing rates are commonly used to classify neurons into putative subtypes [74,82,87], inferring physiological properties of neurons from extracellular recordings is highly problematic as waveform features vary as a function of recording location relative to the morphology of the neuron [88–90]. Even so, we did observe consistent differences in extracellular waveform shapes between ASYN and GFP animals that encourage future investigations. Specifically, we found that WLR and WHR neurons showed a large positive extracellular ‘rebound’ following the initial trough (Fig 6A). This was determined by first normalizing the waveform of each neuron such that the trough = −1 and baseline was zero. Consequently, positive values indicate proportional changes above baseline. The “Rebound Peak” for each neuron was measured as the normalized amplitude of the largest peak following the time of the trough (t = 0 ms). Values for each neuron type are shown in Fig 6B for the GFP and ASYN groups. Rank-sum tests were performed, and significant between-group differences were identified in the WLR and WHR groups (p_WLR_ = 0.003, p_WHR_ = 0.0002 after Holm-Bonferroni correction). See also S9 File. Fig **Supplement to** Fig 6 **individual waveforms.**

Given that the cell-type classification method used here includes two measures of spike width (half width and trough-to-peak), it was conceivable that Rebound Peak was only a second-order indicator of spike-width. To address this, we performed a follow-up analysis that ignored neuron category. Instead, this approach only used waveform width and Rebound Peak to determine which were significant predictors of group (GFP vs. ASYN). This was accomplished using a logistic regression (fitglm) performed on all neurons where group was the outcome variable and waveform half width, peak to trough width, and Rebound Peak were predictors. Of the three measures, only the Rebound Peak was a significant predictor of group (p_overall_ = 0.0022, p_rebound peak_ = 0.017, slope _rebound peak_ = 2.61, n = 185 neurons).

### 3.6. No observed correlation between song features and neuronal firing properties

Next, we investigated whether ASYN and GFP birds differed in their song features and if variation in mean WLR neuronal activity in each bird correlated with song features. Analysis of syllable-level features failed to identify any between-group difference (Mann-Whitney U test, p > 0.05, S2 File. **Fig Exemplar song motif and song syllable data,** S1 File. **Table 1 song data and statistics**).

Investigation of whether firing rates correlated with song features was performed by first calculating the mean firing rate of WLR neurons (the largest group) for each bird. Two birds had to be excluded from the analysis as no WLR neurons were identified in these animals. Mean WLR firing rates were used as the outcome variable for a generalized linear model where the independent variables were song features (mean duration, amplitude, and entropy and CV duration, amplitude, and entropy) and the group (ASYN or GFP). No significant relationship between song features and firing rate was identified (F(10,2) = 11.7, p = 0.082; see S3 File. **Fig No correlation between song and firing rate**).

## 4. Discussion

The goal of this study was to obtain new information about the cellular and network-level activity affected by overexpression of human a-syn protein in an adult male zebra finch model. To carry out this goal, we performed extracellular recordings from song nucleus Area X of the basal ganglia in anesthetized adult male finches and identified five distinct cell types. Our main finding was that wide, low firing rate (WLR) neurons, show reduced firing activity and enhanced post-inhibitory rebound in the a-syn overexpression condition compared to controls. One interpretation is that these WLR neurons are MSNs, and if so, then their activity would align with our working hypothesis that abnormally elevated a-syn protein levels in Area X will decrease MSNs firing rate. However, given the abundance of cell types recorded in Area X of singing finches [74,78], it is necessary to validate the identity of the WLR neurons further. To evaluate the specific role of a-syn expression in MSNs, future directions in this research will employ a custom-made AAV containing a CAMKII promoter driving *SNCA.* This promoter has been previously used in naïve finches to evaluate firing activity of MSNs [91]. We also predicted that GP-projecting neurons would show reduced firing in Area X. VHR neurons may represent these GP-projecting neurons, but they did not show a difference in firing rate between the ASYN and GFP control groups. This finding may reflect low statistical power given the small number of VHR neurons recorded. Future experiments using slice *in vitro* recording methods are needed to confirm WLR cells are MSNs and that VHR neurons as GPs and their firing rate responses to viral manipulation. Intriguingly, a loss of MSNs and therefore, reduced inhibitory synapses onto GPs, occurs in Area X of singing finches virally expressing the Huntington’s Disease gene mutation [92], providing support for our model (Fig 1B) that dampening of MSNs activity leads to increased GP activity and inhibitory modulation of thalamus.

The observed reduction in WLR firing rates observed in Area X of the ASYN finches contrasts with some measures of MSN activity reported in human PD patients and primate PD models. For example, striatal projection neurons in the human basal ganglia of PD patients undergoing deep brain stimulation had high frequency neuronal firing patterns resembling the advanced parkinsonism of the Non-Human Primate model, with hyperactivity and “bursty” neuronal firing activity [14,64]. Future recordings in singing, ambulatory finches with the ASYN phenotype will determine if hyperactivity of MSNs exists and classify the other cell-types and how these various sub-components contribute to changes in real-time song output.

The reduced firing rates of WLR neurons in ASYN expressing finches may reflect reduced mean frequency of spontaneous excitatory postsynaptic glutamatergic currents from cortical song nucleus lMAN onto Area X MSNs and GP neurons. Striatal recordings from h*SNCA* overexpressing mice show reduced glu currents [12]. A recent study in a mouse model of pre-formed a-syn fibrils found that a-syn aggregates in spiny projection neurons decreased evoked corticostriatal glutamate release and induced loss of these synapses [93]. Future work using electrophysiological recordings from Area X brain slices *in vitro* can determine whether glu input is perturbed in our ASYN condition and the impact on synapses.

ASYN-driven abnormal changes in synaptic input to Area X may also be accompanied by altered intrinsic membrane properties. Both the WLR and WHR neuronal classes showed greater positive rebound potential following the initial spike in the ASYN expressing group compared to GFP controls (Fig 6). Under constrained conditions, that shape of the action potential measured from extracellular electrodes can be interpreted as the negative first derivative of intracellular measurements [94]. That said, many additional factors may influence the extracellular potential such as cell morphology, extracellular electrode position, and channel densities [94,95]. Under the assumption that the waveform is indeed proportional to the negative first derivative of intracellular measures, the larger post-peak rebound observed in the ASYN group suggests a stronger rate of hyperpolarization. This increase could contribute to the reduced firing rates observed in the ASYN group (**Fig 4**). To speculate, the rate of hyperpolarization is partly regulated by K^+^ currents, and several classes of K^+^ channels are implicated in altered neuronal excitability in PD models [96]. Patch-clamp electrophysiology in brain slices is a necessary approach to confirm true fluctuations in membrane properties during the post-peak rebound period. Changes in rebound-associated conductance could result from increased SK/BK channel activity [97,98] or a reduction in I_h_ current [99,100], leading to greater hyperpolarization following spikes and dampened excitability. In nigral DA neurons, abnormal a-syn oligo aggregates increase the conductance of K^+^_ATP_ channels leading to reduced firing [101]. In our finch model, a-syn overexpression leading to a-syn aggregate formation in neurons per Bjork et al. (2025) could progressively decrease intrinsic excitability in the WLR neurons via these channels, and/or others. For example, fast-activating voltage-gated Kv3 channels that have been identified in cortical song nucleus RA reduce the spike half-width and allow for more rapid firing [102]. Shaker K^+^ channels are implicated in the firing of RA projection neurons [103]. Higher mRNA expression within Area X has been found for two specific K+ channels, *KCNT2* and *KCTD12*, compared to surrounding tissue [104], but the biophysical properties of these channels require investigation. How a-syn protein pathology as detected in Area X neurons could interfere with channel function, leading to reduced MSNs firing in our model, is not known. By selectively virally overexpressing a-syn in these putative MSNs such as by use of a CAMKII promoter, we can evaluate the relationship between the extent of protein pathology, channel activity, and the overall circuit level activity changes underlying song. In a recent publication, we found that regional changes in virally-expressed human a-syn protein within Area X correlated with changes in syllable level features. For example, we detected a positive correlation between right hemisphere a-syn proteinopathy and a reduction in the variation of harmonic syllable duration, among other features, correlated to the right hemisphere Area X [46]. Our neural recordings here are also from right hemisphere Area X; the contribution of the left Area X is not known. Prior studies suggest a transition in the unilateral activation of the hemispheres: right-side dominance of song nucleus HVC is detected in adult birdsong [105] while the left-side dominates during juvenile learning [106]. Thus, when assessing cell-type changes in electrical activity within brain nuclei aligned with real-time singing behavior, factors to take into account include the recording hemisphere, the distribution of protein expression, and the presence of pathology, e.g., a-syn aggregates in individual cell types.

A benefit of anesthetized recordings is that they allow for high data throughput, enabling the recording of a substantial proportion of neurons without confounds due to animal movement. A limitation of this approach in our finch model is that on-going real time modification of neural activity related to song production cannot be evaluated. It is also likely that neuronal activity was dampened by isoflurane anesthesia, complicating direct comparisons between these data and responses identified in awake finches. While information is not available on the effects of isoflurane on *in vivo* recordings from the striatum of naïve finches or rodents, patch clamp studies in rat brain slices show that brief isoflurane exposure can dampen sodium currents in hippocampal neurons [107] and reduce spiking and bursting modes in thalamic neurons [108]. Our future investigations will compare isoflurane-induced anesthesia on Area X of naïve finches versus awake, singing finches where we can examine how a-syn modulates neuronal dynamics in different cell types under more physiologically relevant conditions.

## 5. Conclusions

Our study takes an important first step forward in evaluating the consequences of a-syn overexpression on the activities of neuronal subtypes involved in vocal behavior. A growing body of research has elucidated the role that neurons in the cortico-striatal-thalamic circuit play in male zebra finch song production. By introducing human a-syn to the zebra finch model, this study supports a systems- and cellular-level investigation of human vocal deficits in PD. This is important given that current medications and brain stimulation used in the treatment of PD fail to resolve the vocal deficits and can worsen them [109–111]. Data from pre-clinical animal models such as the songbird can provide important mechanistic insight needed for the development of new brain-targeted cellular therapies for the vocal dysfunction.

## Supporting information

S1 File Table 1. song data and statistics. For further information, see legends embedded into the tabs of this excel file.
(XLSX)

S2 File Fig Exemplar song motif and song syllable data. (DOCX)

S3 File Fig No correlation between song and firing rate. (DOCX)

S4 File Fig Individual waveforms and autocorrelograms. (DOCX)

S5 File Fig Waveforms, firing rates, and classification. (DOCX)

S6 File Fig Cluster separation between neuronal categories. (DOCX)

S7 File Fig Individual animal variance. (DOCX)

S8 File Fig Rasters for cell types sorted by LV. (DOCX)

S9 File Fig Supplement to Figure 6 individual waveforms. (DOCX)

S10 File Matlab code for ephys. This zip folder contains the matlab files used by Co-Author Cowen to analyze the electrophysiological recordings.
(ZIP)
