# Supplementary material for: Alpha-synuclein overexpression reduces neural activity within a basal ganglia vocal nucleus in a zebra finch model
Source: PLoS One. 2026 Jul 16;21(7):e0333158. doi: 10.1371/journal.pone.0333158 (PMC13374917; doi:10.1371/journal.pone.0333158)
Supplement: S2 File — (DOCX) [file pone.0333158.s002.docx]

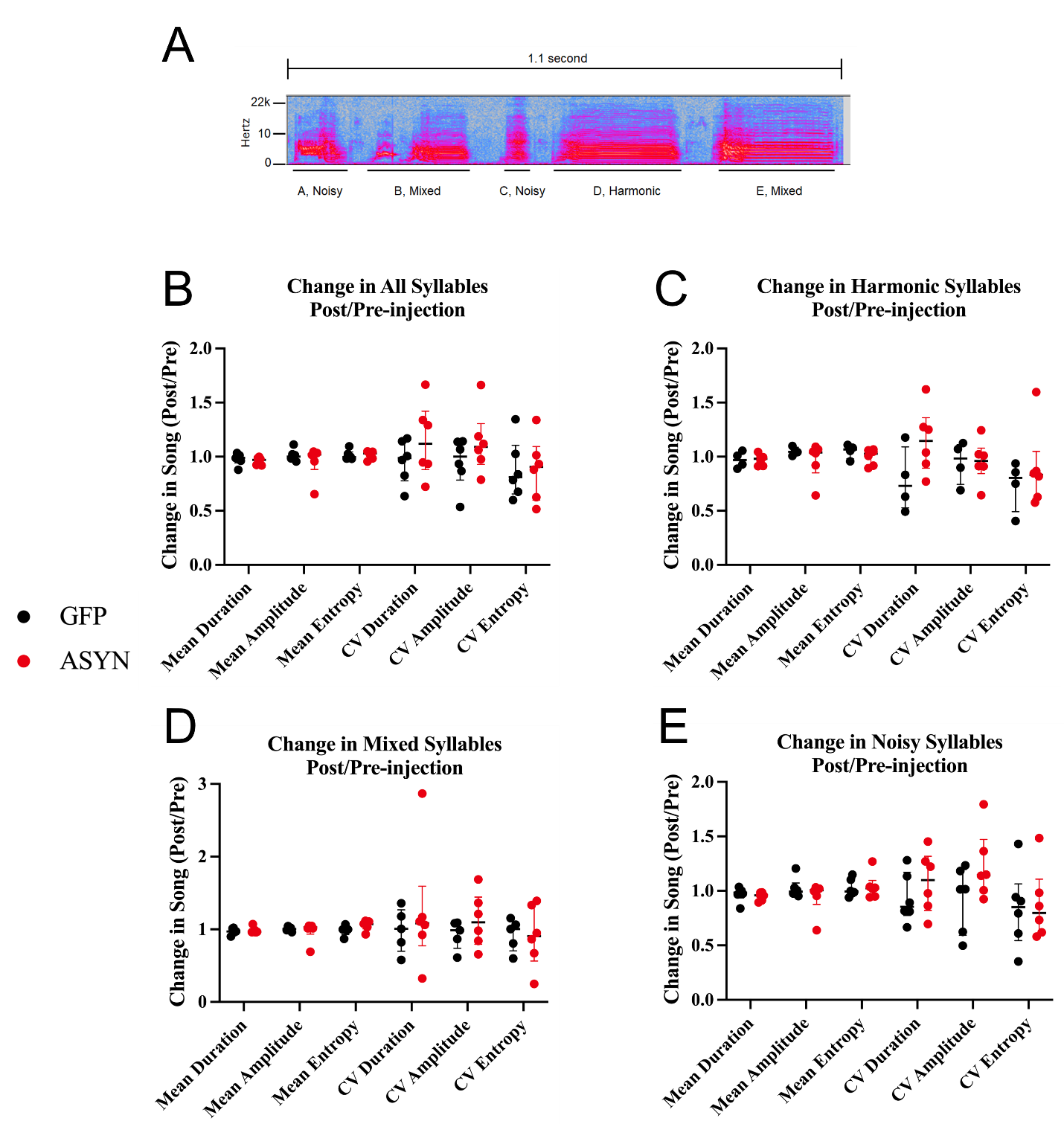


**S2 File. Fig Exemplar song motif and song syllable data.** **A)** Spectrogram of song motif from a pre-injected GFP control bird (as in **Fig 2A-C**) with time in seconds on the x-axis and frequency in kilo (k) Hertz on the y-axis. Unique syllables are assigned a letter (A-E) and classified into harmonic, noisy, or mixed syllables (Badwal A, Poertner J, Samlan RA, Miller JE. Common terminology and acoustic measures for human voice and birdsong. Journal of Speech, Language, and Hearing Research. 2018;61(12)). **B-E)** The mean and SEM scores shown for syllable level acoustic features (duration, amplitude, and entropy) for the ASYN (red) and GFP (black) control groups. Raw scores can be found in the **S1 Table**. The y-axis is the normalized score calculated from dividing the post-AAV injection scores by the pre-injection scores. Each dot represents an average score per finch. **B)** Comparisons of all syllable types combined or by individual syllable types **(C-E)** reveal no group differences (Mann Whitney U, p>0.05). Individual variation in CV scores is detected.
