# Supplementary material for: Alpha-synuclein overexpression reduces neural activity within a basal ganglia vocal nucleus in a zebra finch model
Source: PLoS One. 2026 Jul 16;21(7):e0333158. doi: 10.1371/journal.pone.0333158 (PMC13374917; doi:10.1371/journal.pone.0333158)
Supplement: S3 File — (DOCX) [file pone.0333158.s003.docx]

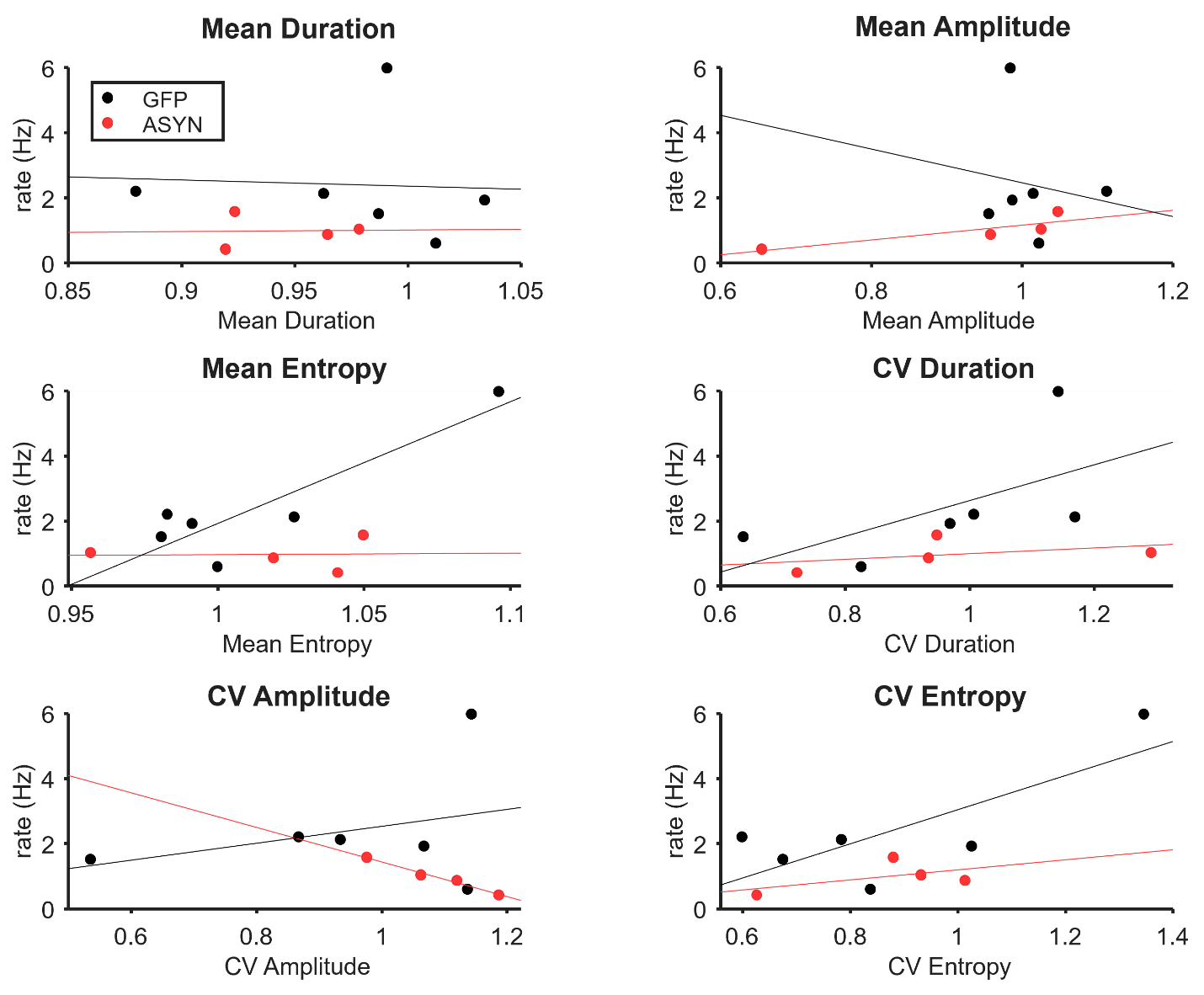


**S3 File. Fig No correlation between song and firing rate.** Scatter plots showing the mean firing rate of Wide-Low-Rate (WLR) neurons averaged for each animal (dot) organized by GFP (black) and ASYN (red) groups. Plots were generated for each song feature, representing all syllable types combined. Generalized linear regression (see Results) did not identify a relationship between firing rate and song features or group (p = 0.0815).
