# Supplementary material for: Alpha-synuclein overexpression reduces neural activity within a basal ganglia vocal nucleus in a zebra finch model
Source: PLoS One. 2026 Jul 16;21(7):e0333158. doi: 10.1371/journal.pone.0333158 (PMC13374917; doi:10.1371/journal.pone.0333158)
Supplement: S4 File — (DOCX) [file pone.0333158.s004.docx]

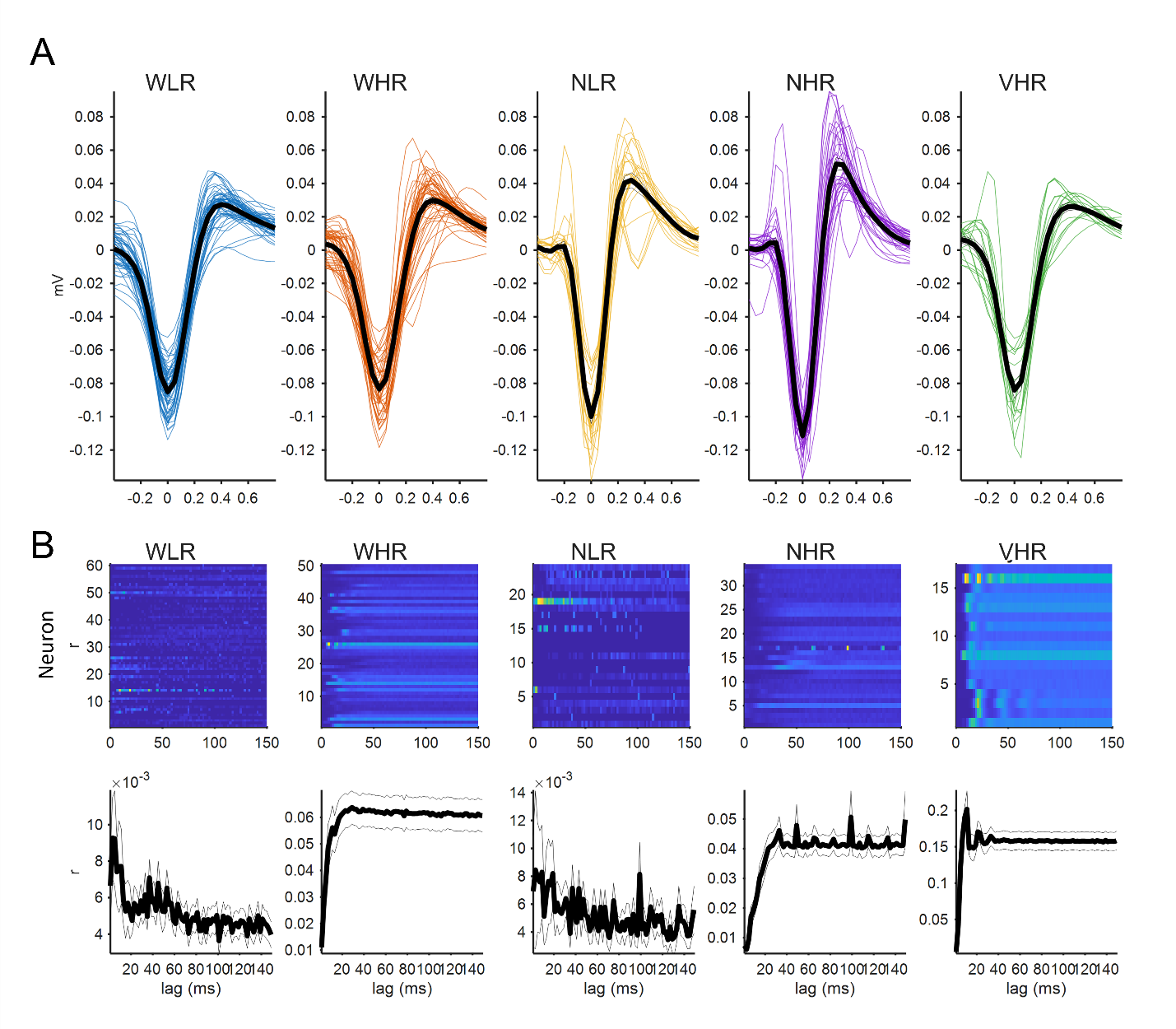


**S4 File. Fig Individual waveforms and autocorrelograms.**  **A)** All individual waveforms for each neuron category are presented. The mean waveform is indicated in black. **B)** Autocorrelograms for all neurons (top). Units are in correlation coefficients (r). The bottom plot shows the means (+/- SEM) of all autocorrelograms. Autocorrelograms and the inter-spike interval (ISI) responses were used to assess the quality of each neuron. Neurons with a large proportion of ISIs below 1.5ms were eliminated from analysis due to potential cross-contamination from other cells. Wide-Low-Rate (WLR), Wide-High-Rate (WHR), Narrow-Low-Rate (NLR), Narrow-High-Rate (NHR), Very-High-Rate (VHR).
