# Supplementary material for: Alpha-synuclein overexpression reduces neural activity within a basal ganglia vocal nucleus in a zebra finch model
Source: PLoS One. 2026 Jul 16;21(7):e0333158. doi: 10.1371/journal.pone.0333158 (PMC13374917; doi:10.1371/journal.pone.0333158)
Supplement: S5 File — (DOCX) [file pone.0333158.s005.docx]

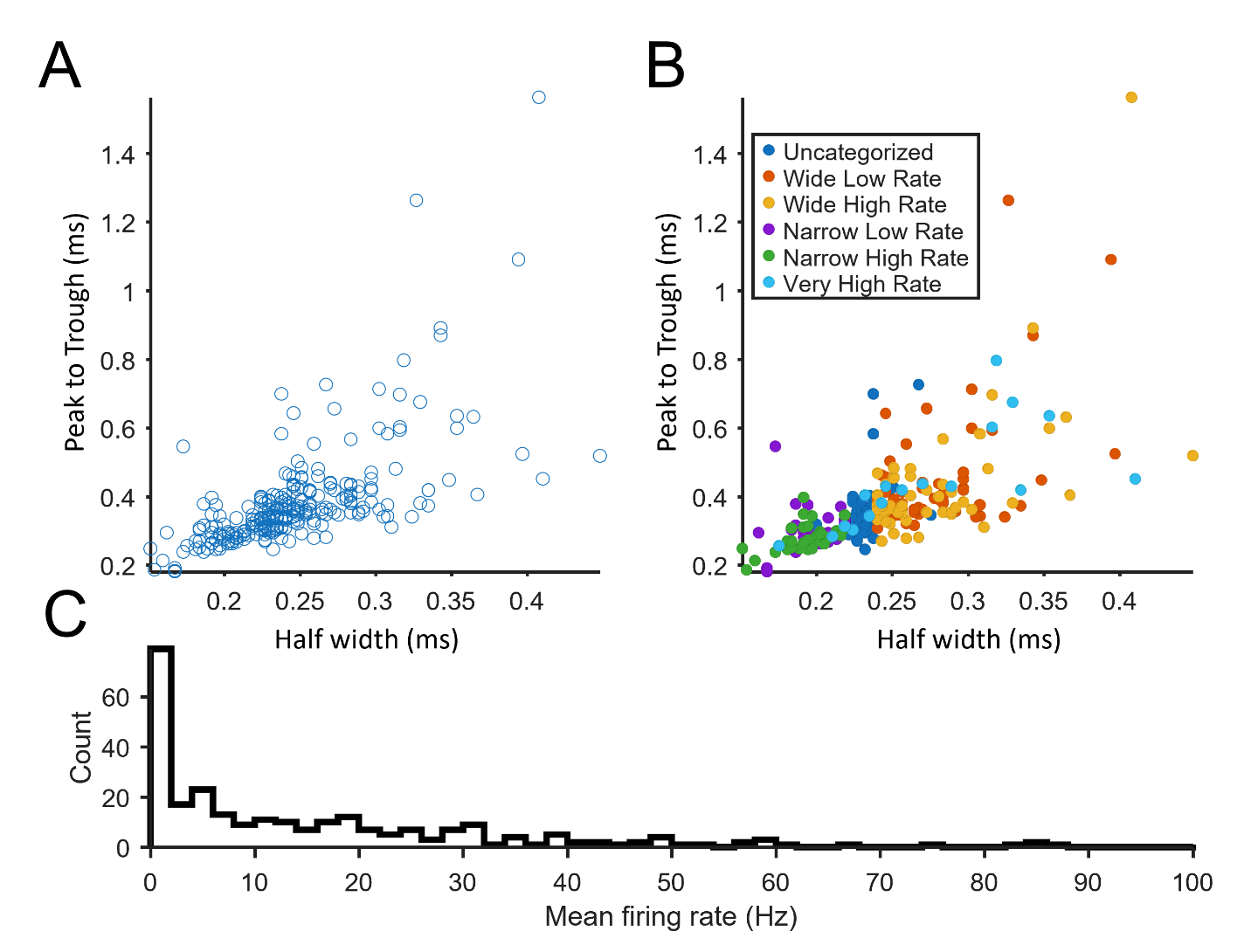


**S5 File. Fig Waveforms, firing rates, and classification. A)** Scatter plot of spike widths for each neuron prior to categorization by cell type. **B)** Same plot but color indicates categorized cell type.  **C)** Histogram of firing rates for all neurons prior to categorization.
