# Supplementary material for: Alpha-synuclein overexpression reduces neural activity within a basal ganglia vocal nucleus in a zebra finch model
Source: PLoS One. 2026 Jul 16;21(7):e0333158. doi: 10.1371/journal.pone.0333158 (PMC13374917; doi:10.1371/journal.pone.0333158)
Supplement: S6 File — (DOCX) [file pone.0333158.s006.docx]

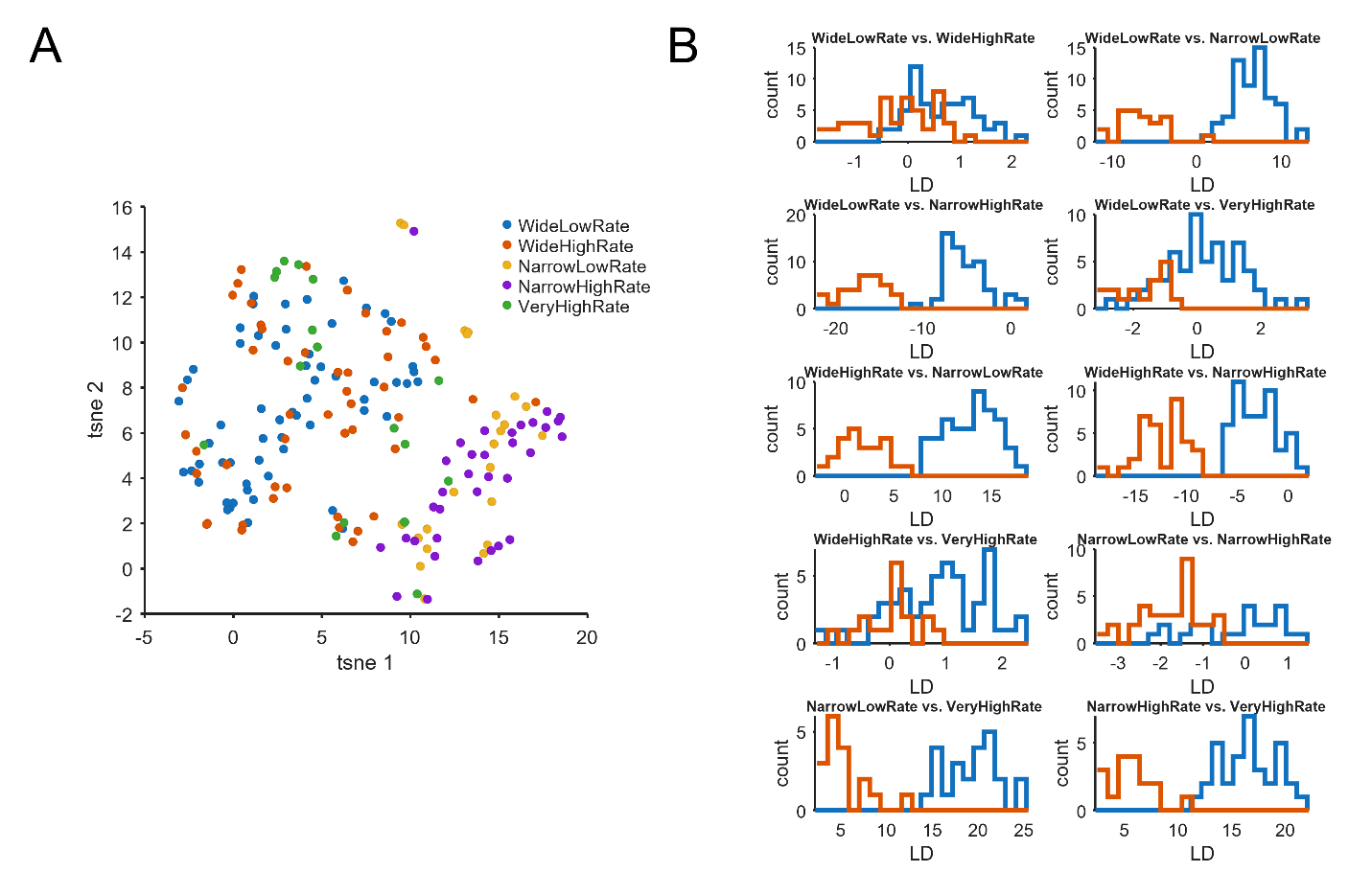


**S6.File Fig Cluster separation between neuronal categories.**

**A)** Visualization of the grouping of the selected categories based on two extracted features derived from waveform shape using tSNE. Based on these two dimensions, narrow waveform cells (purple, yellow) separated from the wide waveform cells. **B)** A more targeted pair-wise comparison of cluster separation based on the entire waveform shape using linear discriminant analysis (LDA). For each pair of selected cells, LDA computed a dimension that could separate the groups. In nearly every pairwise comparison, there is a clear separation between clusters, suggesting that features of the waveform did a reasonable job of separating the different cell types.
