# Supplementary material for: Alpha-synuclein overexpression reduces neural activity within a basal ganglia vocal nucleus in a zebra finch model
Source: PLoS One. 2026 Jul 16;21(7):e0333158. doi: 10.1371/journal.pone.0333158 (PMC13374917; doi:10.1371/journal.pone.0333158)
Supplement: S7 File — (DOCX) [file pone.0333158.s007.docx]

**
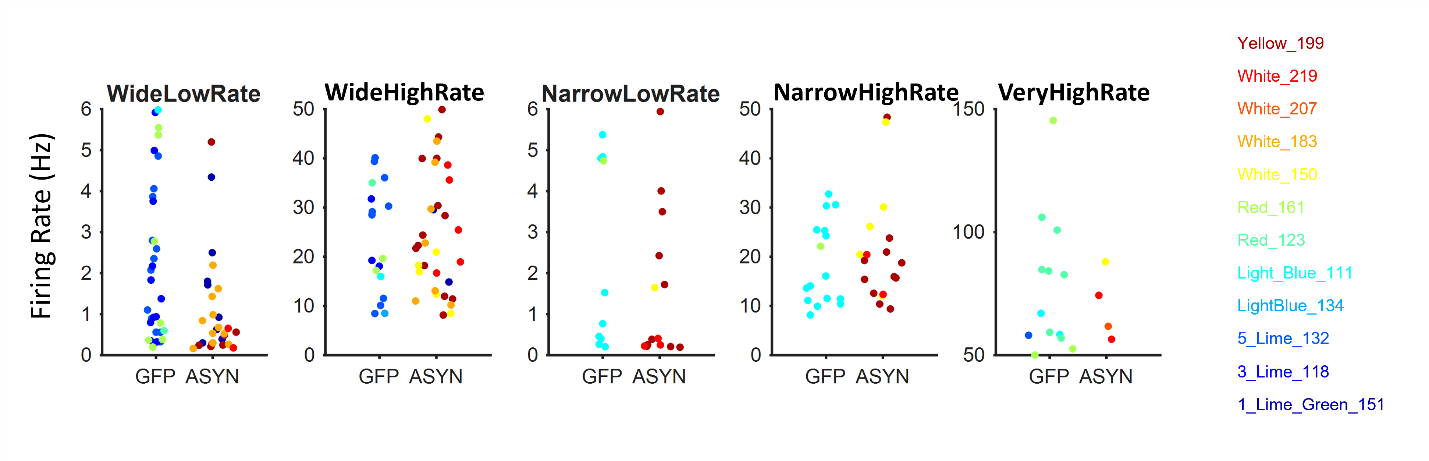
**

**S7 File. Fig Individual animal variance.** This plot corresponds to the data shown in Figure 4C but indicates individual bird ID by color allowing visualization of the within-animal variance. The legend indicates the individual bird ID. This plot presents between-group comparison (GFP vs. ASYN) for each of the five neuron types. Firing rates for WLR neurons were lower in the ASYN group relative to the GFP control (rank-sum test, p = 0.005 prior to correction, p = 0.026 after Holm correction).
