# Supplementary material for: Alpha-synuclein overexpression reduces neural activity within a basal ganglia vocal nucleus in a zebra finch model
Source: PLoS One. 2026 Jul 16;21(7):e0333158. doi: 10.1371/journal.pone.0333158 (PMC13374917; doi:10.1371/journal.pone.0333158)
Supplement: S8 File — (DOCX) [file pone.0333158.s008.docx]

**
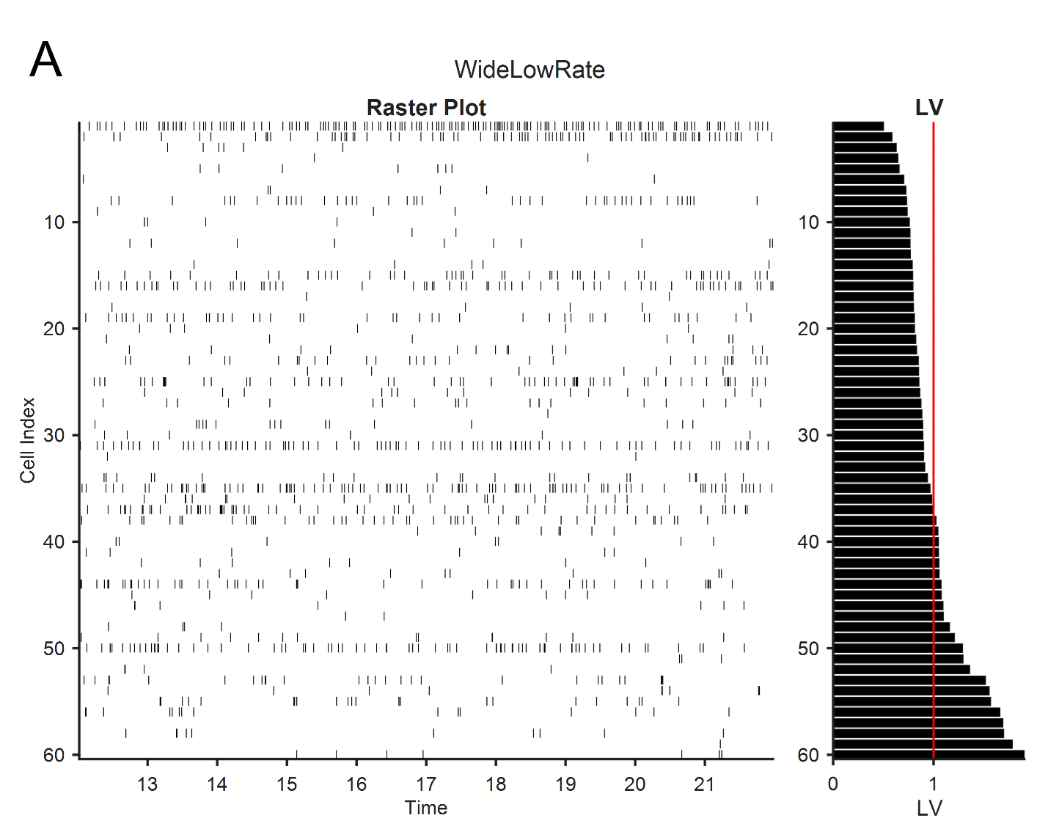
**

**
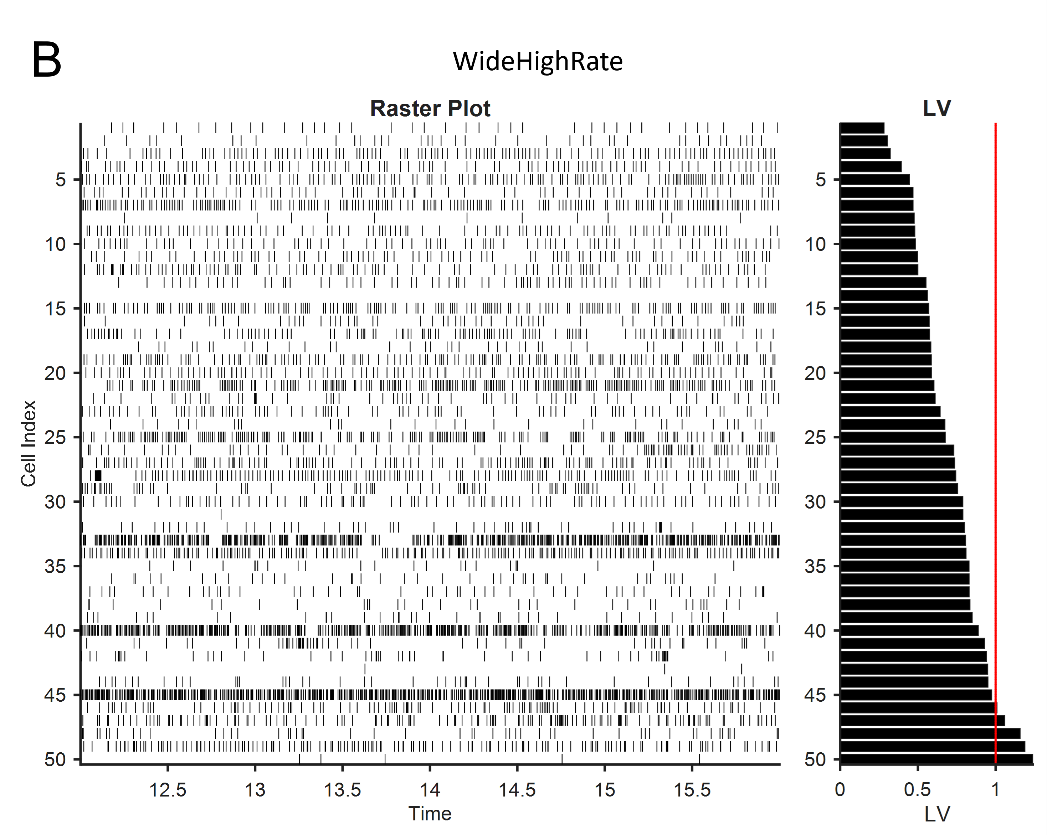
**

**
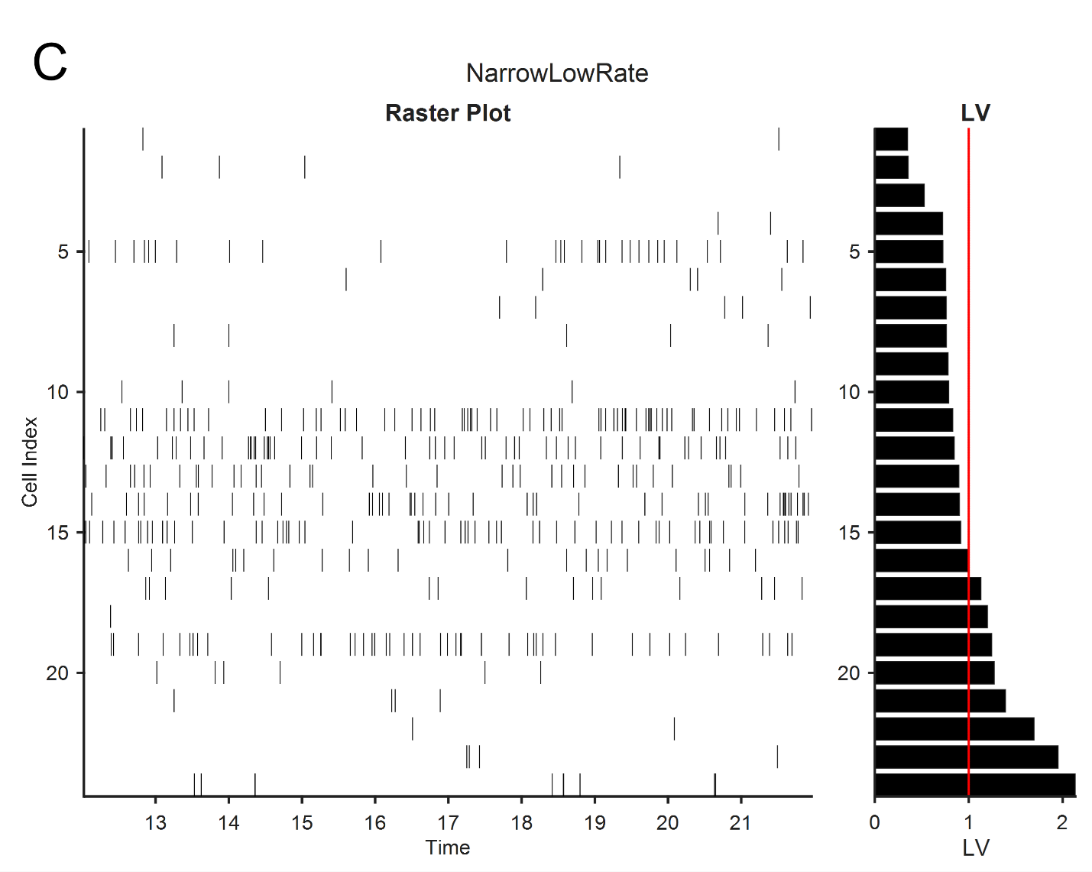
**

**
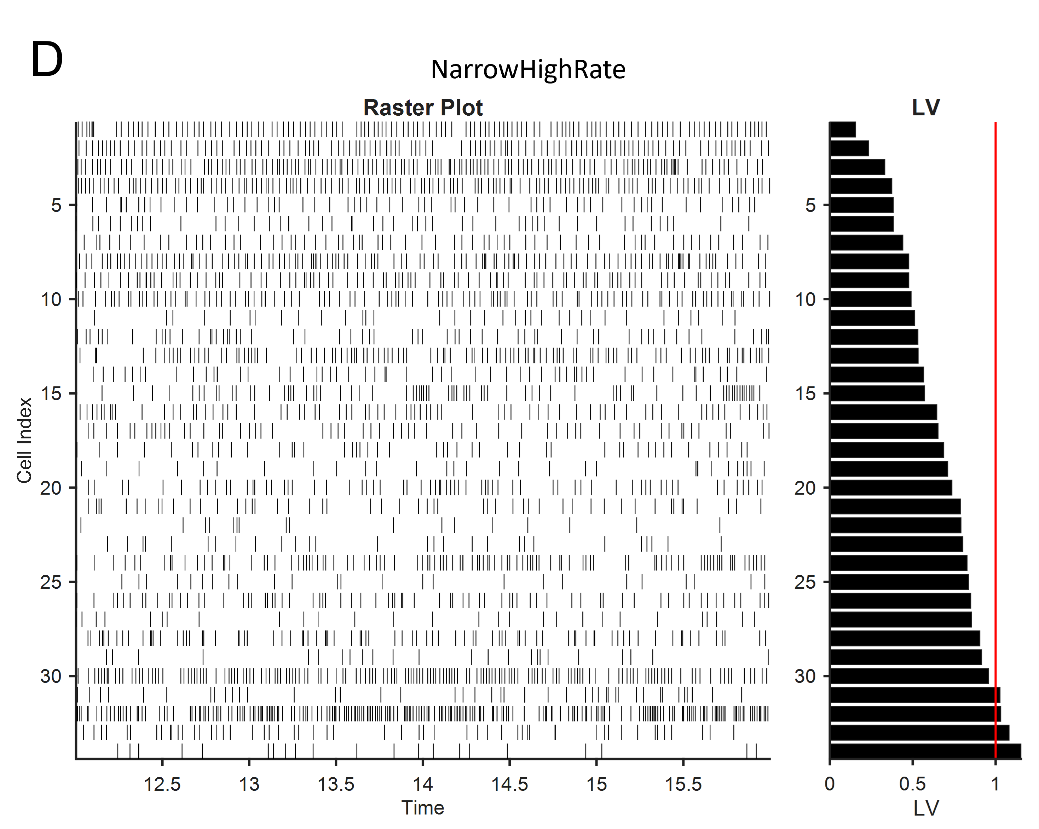
**

**
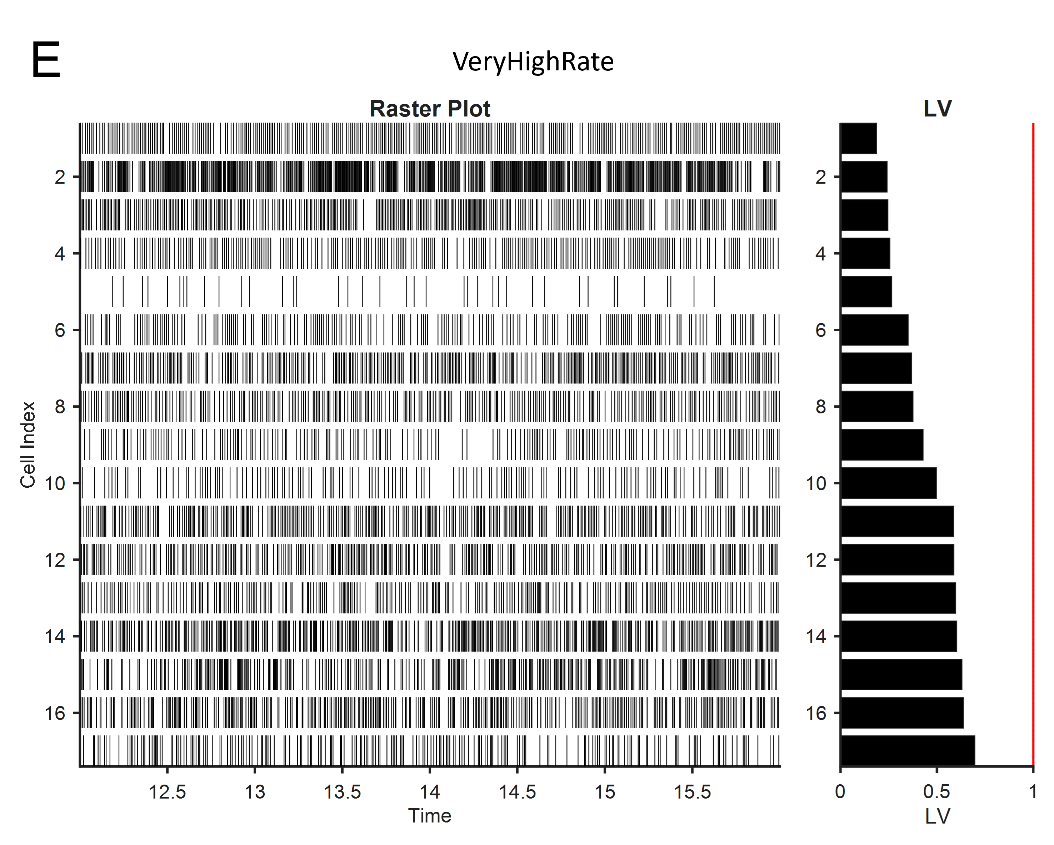
**

**S8 File. Fig Rasters for cell types sorted by LV.** Left: Rastergram of action potentials for each cell of a given type (title) sorted by local variance (LV). Right: The bar plot shows the local variance (LV) for each neuron. The red line indicates LV = 1.0, the accepted threshold for ‘bursting’. **A)** Wide Low Rate; **B)** Wide High Rate; **C)** Narrow Low Rate; **D)** Narrow High Rate; **E)** Very High Rate.
