# Supplementary material for: Alpha-synuclein overexpression reduces neural activity within a basal ganglia vocal nucleus in a zebra finch model
Source: PLoS One. 2026 Jul 16;21(7):e0333158. doi: 10.1371/journal.pone.0333158 (PMC13374917; doi:10.1371/journal.pone.0333158)
Supplement: S9 File — (DOCX) [file pone.0333158.s009.docx]

**
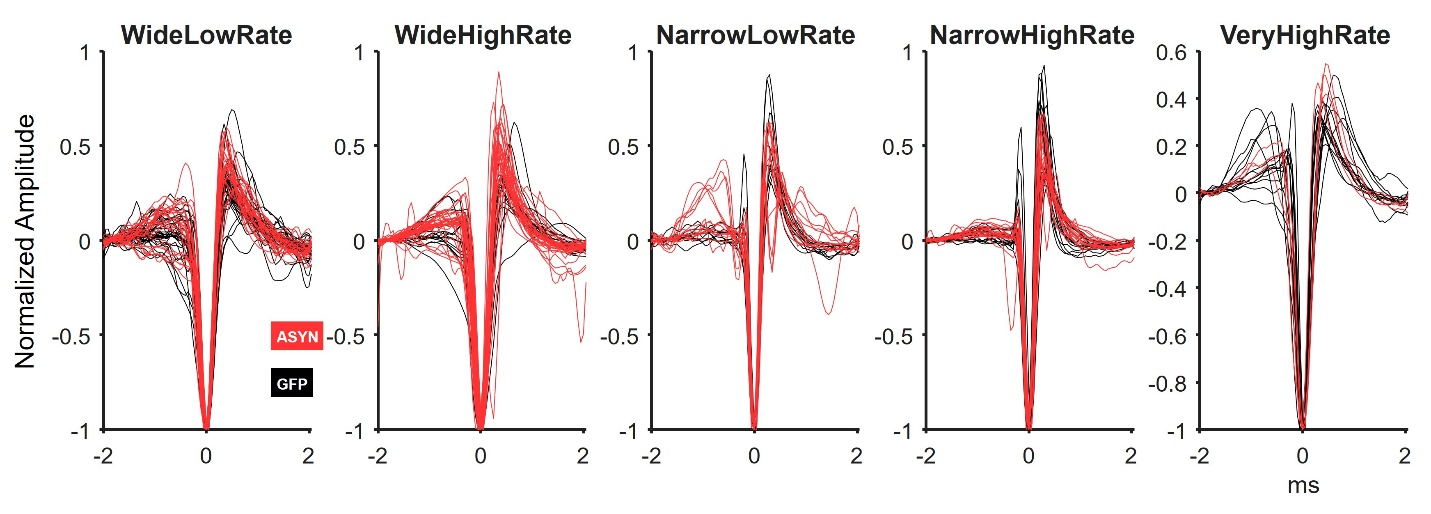
**

**S9 File. Fig Supplement to Figure 6 individual waveforms.** Plots show all individual waveforms that were used for the analyses presented in Figure 6 with the group indicated by color (ASYN vs. GFP control). Units are normalized such that the trough = -1.
